# The Musical Abilities, Pleiotropy, Language, and Environment (MAPLE) Framework for Understanding Musicality-Language Links Across the Lifespan

**DOI:** 10.1162/nol_a_00079

**Published:** 2022-12-16

**Authors:** Srishti Nayak, Peyton L. Coleman, Enikő Ladányi, Rachana Nitin, Daniel E. Gustavson, Simon E. Fisher, Cyrille L. Magne, Reyna L. Gordon

**Affiliations:** Department of Otolaryngology – Head & Neck Surgery, Vanderbilt University Medical Center, Nashville, TN, USA; Department of Psychology, Middle Tennessee State University, Murfreesboro, TN, USA; Vanderbilt Brain Institute, Vanderbilt University, Nashville, TN, USA; Vanderbilt Genetics Institute, Vanderbilt University Medical Center, Nashville, TN, USA; Department of Medicine, Vanderbilt University Medical Center, Nashville, TN, USA; Language and Genetics Department, Max Planck Institute for Psycholinguistics, Nijmegen, The Netherlands; Donders Institute for Brain, Cognition and Behaviour, Radboud University, Nijmegen, The Netherlands; PhD Program in Literacy Studies, Middle Tennessee State University, Murfreesboro, TN, USA; Curb Center for Art, Enterprise, and Public Policy, Vanderbilt University, Nashville, TN, USA; Vanderbilt Kennedy Center, Vanderbilt University Medical Center, TN, USA; Department of Linguistics, Potsdam University, Potsdam, Germany; Vanderbilt University School of Medicine, Vanderbilt University, TN, USA; Institute for Behavioral Genetics, University of Colorado Boulder, Boulder, CO, USA

**Keywords:** complex trait genetics, musicality, speech and language development, pleiotropy, individual differences, neural endophenotypes

## Abstract

Using individual differences approaches, a growing body of literature finds positive associations between musicality and language-related abilities, complementing prior findings of links between musical training and language skills. Despite these associations, musicality has been often overlooked in mainstream models of individual differences in language acquisition and development. To better understand the biological basis of these individual differences, we propose the Musical Abilities, Pleiotropy, Language, and Environment (MAPLE) framework. This novel integrative framework posits that musical and language-related abilities likely share some common genetic architecture (i.e., genetic pleiotropy) in addition to some degree of overlapping neural endophenotypes, and genetic influences on musically and linguistically enriched environments. Drawing upon recent advances in genomic methodologies for unraveling pleiotropy, we outline testable predictions for future research on language development and how its underlying neurobiological substrates may be supported by genetic pleiotropy with musicality. In support of the MAPLE framework, we review and discuss findings from over seventy behavioral and neural studies, highlighting that musicality is robustly associated with individual differences in a range of speech-language skills required for communication and development. These include speech perception-in-noise, prosodic perception, morphosyntactic skills, phonological skills, reading skills, and aspects of second/foreign language learning. Overall, the current work provides a clear agenda and framework for studying musicality-language links using individual differences approaches, with an emphasis on leveraging advances in the genomics of complex musicality and language traits.

## INTRODUCTION

Language and music are ubiquitous forms of communication across the world (Ujfalussy, 1993), and both make use of certain essential acoustic and perceptual parameters that facilitate effective expression (Jackendoff, 2009; Molino, 2000; Patel, 2003). For example, the ability to perceive **rhythm** (key terms are defined in the Glossary within the Supporting Information at https://doi.org/10.1162/nol_a_00079) is important in both musical and language contexts (Besson & Schön, 2012; Slevc, 2012). Research has also shown an overlap in the cognitive and neural processes that are recruited during musical and language-related tasks (Fedorenko et al., 2009; Kotz et al., 2018; Kunert et al., 2015; Merchant et al., 2015). Based on the overlaps and dissociations in musical and language processing, many researchers have highlighted the functional relationships between **musicality** and language within cognitive and neural frameworks (Gordon & Magne, 2017; Jantzen et al., 2016; Patel, 2008).

The majority of studies have used one of the four following approaches to map associations, overlaps, or similarities between musicality and language: (a) musical training or intervention studies (for reviews, see Tierney & Kraus, 2013; White et al., 2013); (b) comparisons between musicians and non-musicians on language-related tasks (for a review, see Coffey et al., 2017); (c) comparisons of neurocognitive processing of linguistic and musical information (Sammler & Elmer, 2020); and (d) work showing the effects of musical priming in improving language task performance (Schön & Tillmann, 2015). Much of our current understanding of musicality-language links comes from these informative study designs; however, these studies largely obscure preexisting individual differences in musical and language traits. Studying individual differences allows us to capture the range of human experiences stemming from musical and language abilities, including social engagement, communication, academic and professional outcomes, and overall quality of life.

While prevailing explanations for stronger musicality-language links in musicians compared to non-musicians are often framed in terms of neuroplasticity or cognitive transfer (e.g., Bidelman & Alain, 2015; Patel, 2014), many authors have pointed out that individuals may self-select into higher levels of musical experience and engagement (i.e., musical training, instrument playing, music listening habits) based on higher levels of musical aptitude (e.g., Schellenberg, 2015). This pattern can be seen from very early in development (e.g., Brandt et al., 2012). For example, both musical aptitude (e.g., music perception skills) and musical engagement (e.g., musical listening, practice, or training) are traits that show high interindividual variation within populations. Further, twin studies have shown that these musicality traits are all moderately heritable—i.e., that genetic variation partly accounts for the observed individual differences (Mosing et al., 2014; Seesjärvi et al., 2016; Ullén et al., 2014). This framing presents a challenge to prior explanations in terms of transfer effects of music on language (Chan et al., 1998; Kraus & Chandrasekaran, 2010; Moreno, 2009; Slater et al., 2015; Tierney & Kraus, 2013). For example, differential language abilities in groups with different levels of musical engagement could be driven by *who* ends up pursuing musical interests, training, or practice.

When considering the human capacity for music and language, it is important to consider variability in musical *skills* beyond variability in musical *training* or *experience*. For example, studies of individual differences demonstrate positive associations between musical rhythm abilities and grammatical skills (Gordon, Shivers, et al., 2015; Lee et al., 2020), reading-related skills (Ozernov-Palchik et al., 2018; Woodruff Carr et al., 2014), prosodic perception (Hausen et al., 2013; Morrill et al., 2015), and speech discrimination (Swaminathan & Schellenberg, 2020). On the other end of the spectrum, impaired musical rhythm abilities are frequently comorbid with language-related disorders (Ladányi et al., 2020). Moreover, some frameworks linking music and language have emphasized the need to understand genetic and developmental factors (Schellenberg, 2020; Zuk & Gaab, 2018), and to account for the potential confounding role of genetics when studying environmental factors shaping development (Hart et al., 2021), such as musical experiences. Given that we only have a nascent understanding of individual differences in these traits, and of how their relationship evolves over the lifespan, it is important to consider other developmental and biological explanations for associations between musical and language traits.

Many traits that show high degrees of interindividual variability, and that fall along a continuous spectrum, are referred to as complex traits in the genetic literature. Complex trait **phenotypes** are polygenic, meaning they are influenced by multiple genes, with effects at each genetic locus contributing a small amount of variance (Crouch & Bodmer, 2020; Watanabe et al., 2019). There is emerging evidence that musicality and language-related traits exhibit polygenicity when investigated in well-powered genomic studies (Doust et al., 2022; Eising et al., 2022; Niarchou et al., 2022). Further, as we will review in more detail below, traits related to musicality and language are often phenotypically associated with each other, i.e., interindividual differences studies reveal significant correlations between tasks performed across the different domains (e.g., Gordon, Shivers, et al., 2015; Morrill et al., 2015; Yu et al., 2017). These correlations allow us to leverage principles from the field of genetics to guide discovery of theorized biological pathways that may be shared between musicality and language traits. In particular, Cheverud’s conjecture posits that distinct traits showing high phenotypic correlations are likely inherited together and influenced by alleles at a common set of genetic loci (Cheverud, 1988). Such biological patterns of shared genetic architecture, termed **pleiotropy**, have been demonstrated in many complex traits (Sodini et al., 2018). Similarly, based on known cross-trait correlations, musicality and language-related traits are likely influenced (at least in part) by genetic variations at the same genes (Bulik-Sullivan et al., 2015; Wesseldijk et al., 2021). Although, note that given their polygenic nature, this does not discount the existence of genetic effects that contribute uniquely to musicality and language traits.

The overarching objective of this work is to present a framework for investigating biological relationships between musical and language traits, in the context of existing behavioral, neural, and genetic evidence for associations between these domains. In this article, we introduce a framework, as well as a set of testable hypotheses, for understanding the biological and environmental mechanisms by which **polygenic pleiotropy** between musicality and speech-language traits can result in observed correlations between musical abilities and language-related abilities. While the environmental effects of music on cognition have traditionally been explained via models of neuroplasticity (e.g., Bidelman & Alain, 2015; Patel, 2014), behavioral genetics findings allow us to reimagine what musical environments can tell us. For example, alongside their influence on musical and language abilities, genes also exert influence on music-related environments (e.g., music practice: Mosing et al., 2014; music instrument engagement: Gustavson et al., 2021).

Further, potential neural **endophenotypes** highlighted from current evidence can help clarify cascading biological mechanisms of musicality-language links across lifespan development. Endophenotypes can be thought of as intermediate biological phenotypes that are functionally involved in the relationship between a genotype and a phenotype of interest (Gottesman & Gould, 2003). For example, measures of basal ganglia neuroanatomy or of prefrontal cortex function could be explored as neural endophenotypes mediating the effects of genetic variants on musicality and language traits. Discovery of **gene expression** patterns in key brain regions and developmental processes of interest (e.g., neocortical development: Miller et al., 2014) could help directly map associations among genetic variants, neural endophenotypes (including gene expression and regulation patterns in the brain), and correlated musicality and language traits (see Kong et al., 2020, for an example of identifying convergence between functional magnetic resonance imaging (fMRI) based language circuitry and gene expression patterns). Like musicality and language traits themselves, neural endophenotypes (including gene expression patterns) are shaped by interacting genetic and environmental influences.

We therefore argue that future research on musicality-language associations should aim to incorporate genetic studies of both musicality and language phenotypes as well as associated neural endophenotypes. We suggest ways in which this can be achieved by leveraging existing data and collaborative efforts. Specifically, genetic/genomic data can be used to (a) understand the shared genetic architecture of musicality and language; (b) disentangle the interplay between genes and environment; and (c) inform predictive models and intervention efforts in the context of language-related disorders, using data available on music-related traits.

### Structure and Scope of the Current Work

The structure of the current work is as follows: First, we provide an overview of **heritability** estimates for musicality and speech-language phenotypes reported by twin and family-based studies, and discuss how these estimates can inform our understanding of correlated individual differences in musical and language abilities. Second, we propose a novel framework for understanding links between musical and language abilities, with a focus on shared genetic architecture and mediating neural endophenotypes. Third, we provide a specific overview of genetics approaches that can be integrated into current investigations of musicality-language links. Fourth, we outline specific genetic predictions that will help to systematically test our framework, and set an agenda for how the field can pursue these directions. Last, we provide supporting evidence for our framework, with bibliometric analyses, and a synthesis of behavioral studies examining associations between musicality-language traits. We also discuss adjacent evidence from the neural correlates of individual differences in musical or language abilities, where these abilities are associated. The literature synthesis is organized by domain of language ability: We discuss research on musicality and speech skills, reading skills, and grammar skills respectively.

We constrain the scope of the current work in a few important ways. One, our discussion and framework focus on individual differences in stable traits. Therefore, studies that investigate priming effects of music on language; language in musically trained and untrained individuals; similarities in neural processing of musical and linguistic stimuli; and effects of musical interventions for language, are considered outside the scope of the current work, while certainly remaining relevant for understanding relationships between musicality and language. Similarly, we do not discuss lines of research focused on musical state-driven modulation of neurobiology (e.g., changes in gene expression after listening to music: Nair et al., 2021), while these studies are informative for different purposes. Two, our framework focuses mainly on the potentially supportive role of musical abilities for language across the lifespan. We also acknowledge that the reverse remains possible, i.e., that language abilities support musical skills (e.g., Roncaglia-Denissen et al.’s, 2016, finding that L2 learners show enhanced musical rhythm). Understanding biological relationships between musicality and language traits (e.g., shared genetic architecture of these skills) will be useful for understanding both musical and language-related outcomes. Three, while we review evidence of the neural correlates of individual differences in musicality-language skills, we generally do not focus on the many important studies that experimentally manipulate processing of musical and linguistic stimuli and report primarily group means in one condition versus another; such designs are not necessarily informative about individual differences in stable musicality and language traits (see Hedge et al., 2018, for extended discussion about trade-offs between stable individual metrics and reliable experimental effects).

## HERITABILITY OF SPEECH-LANGUAGE AND MUSICALITY PHENOTYPES

Thus far, the genetic architecture of musicality and language has been primarily studied separately, through twin and family-based methods that inform us about the heritability of specific traits. For example, twin studies show that many speech-language traits are moderately heritable, as summarized in Table 1. Similarly, twin and family-based studies show that musical abilities (e.g., pitch and rhythm sensitivity) have a significant genetic component (Drayna et al., 2001; Seesjärvi et al., 2016; Ullén et al., 2014), as does musical engagement, including accomplishment and training (Hambrick & Tucker-Drob, 2014), as summarized in Table 2. Emerging evidence also finds that both musical aptitude and engagement are genetically associated with language-related traits such as verbal ability (Gustavson et al., 2021; Wesseldijk et al., 2021).

**Table 1.** Heritability estimates of speech-language and reading-related phenotypes.

**Language domain**	**Construct definition**	**Measure description**	**Heritability estimates**	**Citations**
***Speech/Language***				
Spoken Language / Oral Skills	Includes measures of word object mapping, semantics (e.g., finding relationships between words), and morphosyntactic skills.	Relational vocabulary (from Test of Language Development, primary, 3rd ed.: TOLD-P:3)	0.40–0.70	Rice et al., 2018
	**Verbal memory:** the ability to recall what has been heard or read.	Story memory; sentence memory; nonword repetition.	0.48–0.87	Samuelsson et al., 2007 (3 samples)
	**Semantics:** includes measures of word object mapping, and understanding conceptual relationships between words.	Grammatic understanding task (from TOLD-P:3).	0.10–0.70	Rice et al., 2018
	**Oral skills composite**	Composite performance on tasks assessing expressive and receptive vocabulary, naming abilities, and oral language skills.	0.34	Andreola et al., 2021 (meta-analysis of 10 studies)
	**Vocabulary size:** receptive and expressive vocabulary size, tested directly or via parent-report checklist (in young children).	Receptive vocabulary skills; picture vocabulary comprehension; parent-reported vocabulary production.	0.18–0.67	Babajani-Feremi, 2017; Dale et al., 2018; Rice et al., 2018; Samuelsson et al., 2007
	**Impaired language achievement** (in absence of other developmental or sensory impairments).	Low performance on receptive language (e.g., vocabulary and grammar), expressive language (e.g., vocabulary, morphosyntax and grammar).	0.45	Tomblin & Buckwalter, 1998
	**Language impairments** with speech-language pathologist (SLP) referral.	Low performance (relative to sample mean) on a language battery, in addition to having received speech language therapy or other speech pathologist services by age 7 yrs.	0.96–0.97	Bishop & Hayiou-Thomas, 2008
Verbal ability	**Verbal fluency:** includes general fluency and semantic-specific fluency components.	Phonemic fluency (how many words can you write beginning with one letter and ending with another in the time limit); semantic fluency (list as many names of things in a category).	0.65–0.80	Gustavson et al., 2019
	**Verbal ability:** tests of overall verbal knowledge, comprehension, and fluency, tested through conceptual tests.	Odd one out tests; synonym tests; vocabulary tests; or verbal fluency tests.	0.60	Gustavson et al., 2021; Wesseldijk et al., 2021
	**Language comprehension**	Story comprehension (the ability to listen to a story or narrative and accurately answer questions about its content, i.e., comprehend it).	0.32	Babajani-Feremi, 2017
Speech production	**Speech articulation:** ability to articulate real consonant sounds in single words and conversational speech, both spontaneously and through imitation.	Goldman-Fristoe Test of Articulation.	0.25–0.60	Rice et al., 2018; Stein et al., 2011
	**Speech abilities**	Low performance (relative to sample mean) on a speech composite consisting of a speech articulation task and a nonword repetition task (also taps phonological working memory).	0.56	Bishop & Hayiou-Thomas, 2008; Hayiou-Thomas, 2008
***Reading***				
Accuracy and speed of reading	**Reading composite**	Combination of letter-word knowledge, phonological decoding, and reading comprehension phenotypes.	0.66	Andreola et al., 2021 (meta-analysis of 48 studies)
	**Letter-word knowledge**: the recognition and identification of how letters form words (called “general reading construct” in paper).	Oral reading recognition; letter/word identification.	0.56–0.62	Andreola et al., 2021 (meta-analysis of 32 studies); Babajani-Feremi, 2017
	**Phonological decoding**: the ability to break written words into sounds or syllables based on the phonemic representations of your language to support word recognition.	Irregular word decoding; phoneme decoding.	0.68	Andreola et al., 2021 (meta-analysis of 13 studies)
	**Reading comprehension**: the ability to understand and process written text.	Text/story comprehension; reading achievement.	0.68	Andreola et al., 2021 (meta-analysis of 32 studies)
Print knowledge	Knowledge about the rules of print.	Left-to-right reading; letter recognition, environmental print exposure; concepts about print.	0.26	Samuelsson et al., 2007
Phonological awareness	Processing speech and phonological/lexical retrieval.	Comprehensive Test of Phonological Processing (CTOPP); syllable and phoneme blending; word elision; syllable and phoneme elision; rhyme recognition; phoneme isolation; phonemic deletion; rhyme recognition; phonemic segmentation.	0.46–0.64	Andreola et al., 2021 (meta-analysis of 13 studies); Samuelsson et al., 2007 (3 samples)
Literacy	Composite performance on standardized reading, writing, speaking, and listening tasks, assessed through teacher evaluations, and testing via telephone and internet.	Reading fluency (reading simple sentences); literal comprehension of sentences; analysis of written text.	0.68–0.77	Kovas et al., 2013
	**Spelling**: the ability to form words with the correct order of letters.	Orthographic decoding, regular/irregular spelling.	0.80–0.91	Andreola et al., 2021 (meta-analysis of 15 studies); Lewis et al., 2018
	**Conversational language productivity**	Length and complexity of children’s utterances in conversation, usually computed as the mean number of morphemes in utterances sampled.	0.17–0.61	Dale et al., 2018; DeThorne et al., 2012
***Grammar***				
Morphosyntactic abilities	Expressive and receptive skills relating to how words are formed and how the order of words results in understandable phrases and clauses. May include tasks where whole sentences need to be accurately recalled, tapping morphosyntax skills.	Sentence complexity; grammatical property of finiteness marking; grammatical knowledge; productive morphology.	0.36–0.92	Bishop et al., 2006; Dale et al., 2000; Rice et al., 2018; Samuelsson et al., 2007
	Low performance on grammatical/morphosyntactic tests, or indicated by tests designed to screen or measure grammatical impairment.	Grammatical property of finiteness marking; third-person singular; past tense obligatory use; receptive grammar.	0.31–1.0	Bishop et al., 1995; Dale et al., 2018; Rice et al., 2018

*Note*. Table shows moderate heritability of speech/language and reading phenotypes. Each heritability estimate provided indicates the percentage of overall variance in the trait that is genetically influenced, as computed by twin and family-based studies cited here. Broad definitions for constructs are provided, and corresponding phenotypic measures corresponding to each speech-language and reading construct of interest are outlined. TOLD-P:3: Test of Language Development, Primary, 3rd ed. (Newcomer & Hammill, 1997). Goldman-Fristoe Test of Articulation, 2nd ed. (Goldman & Fristoe, 2000). CTOPP: Comprehensive Test of Phonological Processing (Wagner et al., 1999).

**Table 2.** Heritability estimates of musicality phenotypes.

**Construct**	**Measure description**	**Heritability estimates**	**Citations**
Rhythm perception	Discriminating between rhythms; determining whether a rhythm is the same as or different than a reference rhythm.	0.5	Mosing et al., 2016; Ullén et al., 2014
Melody perception	Discriminating between melodies; identifying which note in a given melody differs from a reference melody.	0.58–0.59	Seesjärvi et al., 2016; Ullén et al., 2014
Pitch perception	Discriminating between pitches; identifying whether a given pitch is higher or lower than a reference pitch.	0.4–0.8	Drayna et al., 2001; Ullén et al., 2014
Rhythm production	Isochronous motor timing (self-paced).	0.34	Mosing et al., 2016
Music practice	Duration and frequency of practicing a musical instrument or singing.	0.41–0.69	Butkovic et al., 2015; Mosing et al., 2014
Music flow	Degree of proneness to experiencing psychological flow while engaging in musical activities.	0.4	Butkovic et al., 2015
Musical talent	Self-reported exceptional musical talent (singing on playing instruments) as measured by a self-reported talent inventory.	0.26–0.92	Hambrick & Tucker-Drob, 2014; Vinkhuyzen et al., 2009
Musical aptitude	Self-reports of average or above average musical aptitude (singing or playing instruments) on a self-reported talent inventory.	0.30–0.66	Vinkhuyzen et al., 2009
	For heritability of specific objectively-measured music aptitude traits (rhythm perception, melody perception, pitch perception), see estimates from Ullén et al. (2014), reported above.		
Musical instrument engagement	Self-reported interest, instruction, and talent with musical instruments.	0.78	Gustavson et al., 2021
Singing	Self-reported interest, instruction, and talent with vocal music (singing).	0.43	Gustavson et al., 2021

*Note*. Table shows moderate heritability of musicality phenotypes. Each heritability estimate provided indicates the percentage of overall variance in the trait that is genetically influenced, as computed by twin and family-based studies cited here. Broad definitions for constructs are provided, and corresponding phenotypic measures corresponding to each musicality construct of interest are outlined.

It is important to distinguish this twin and family-based evidence from other types of genetic investigations that focus on altered music and/or language skills in the context of genomic abnormalities affecting a single gene or chromosomal location, such as in individuals with Williams, Prader-Willi, or Angelman syndromes, or in families with FOXP2 disruptions. While ongoing work on these disorders (e.g., Williams syndrome: Kasdan, Gordon, et al., 2022; Thakur et al., 2018; Prader-Willi and Angelman syndrome: Kotler et al., 2019; Mehr et al., 2017; FOXP2 disruptions: Alcock et al., 2000; Fisher & Scharff, 2009) can help to reveal causal pathways linked to rare genetic variation at particular loci, our goal here is to outline steps for understanding the potentially overlapping genetic architecture between musicality and language traits as it relates to common genetic variation in the broader population, which will likely require a genome-wide perspective.

Genome-wide investigations can help clarify mechanisms by which shared genetic architecture between musicality and language-related traits may result in associations observed at the phenotypic level. Further, increased access to high-throughput genetic data collected in large cohorts, coupled with meta-analytical efforts, has greatly improved the potential for understanding the overlapping biology of distinct complex traits related to musicality and speech-language abilities. For example, current statistical genetics methods make it possible to test for **genetic correlations** between traits even when genetic samples and phenotypic measures have been collected in *separate* samples (Bulik-Sullivan et al., 2015). Genetic correlations inform us about the degree of phenotypic covariance in two traits explained by shared genetic variation (Watanabe et al., 2019; Weissbrod et al., 2018).

## THE MAPLE FRAMEWORK FOR CLARIFYING BIOLOGICAL RELATIONSHIPS BETWEEN MUSICALITY AND LANGUAGE ACROSS THE LIFESPAN

The earliest stages of spoken language development (e.g., in infancy) involve learning to segment speech, recognizing vowel and consonant contrasts, and parsing rhythmic patterns in one’s native language(s), which all highly overlap with musical listening skills (Brandt et al., 2012; c.f. signed language development). As Brandt et al. (2012) argue, the listening skills of very young developing humans (fetuses, newborns, and infants included) are attuned to features of spoken language that are also crucial features of musical listening (e.g., pitch, timbre, rhythm, dynamic stress), which form the basis of effective speech processing and lay the foundation for language learning over the course of development (Sanchez-Alonso & Aslin, 2022). The important role that processing these cues plays in language development is further evidenced by much greater than chance prevalence of co-occurring cases of musical and language impairments in children (Ladányi et al., 2020; Peretz & Vuvan, 2017). In particular, children with disorders of language (e.g., developmental language disorder: DLD) and reading (e.g., dyslexia) have higher prevalence of rhythm and melody impairments than age-matched typically developing peers, providing evidence of biological relationships between these deficits (Ladányi et al., 2020). As reviewed in the current article, bountiful supporting evidence shows correlations between musicality and language skills in typically developing children and adults, including associations for multiple levels and domains of language processing (speech, reading, vocabulary, and grammar).

**Figure 1.**
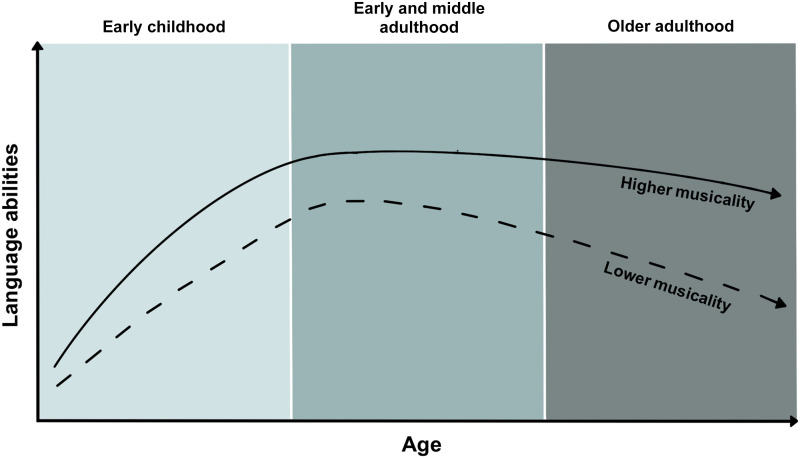
Schematic illustration of the proposed role of musicality in language across the lifespan, showing the curvilinear relationship of language abilities across ages, moderated by relatively higher and lower levels of overall musicality (e.g., musical abilities, engagement, and environments). We propose that individuals with relatively higher musicality on a spectrum of typical individual differences (solid curved line) will have enhanced language abilities and/or steeper developmental trajectories in early stages of life compared to those with relatively lower musicality, or impairments in musical abilities (dashed curved line). Similarly, we propose that in adulthood and in the context of aging, those with relatively higher musicality will experience extended maintenance of peak performance, and slower decline in speech-language function later in life (e.g., efficiency of their speech perception in noise).

These avenues of research have clinical significance in addition to basic science value. Just as musicality might be leveraged for early identification of risk for language-related disorders, musical abilities can help account for resilience to speech-language problems in a variety of common conditions of older adulthood (e.g., dementia, Alzheimer’s disease, age-related hearing loss). For example, shared musicality and language-related genetic architectures could also be implicated in inner ear function, neuroplasticity, and other adaptive mechanisms in the context of brain damage, or natural brain aging, such that those with genetic predispositions for enhanced musical abilities might also have genetic resilience to the magnitude or rate of neurodegeneration associated with language-related problems. Figure 1 illustrates the relationship between individual differences in musicality and language abilities, as we broadly envision it playing out across the lifespan.

**Figure 2.**
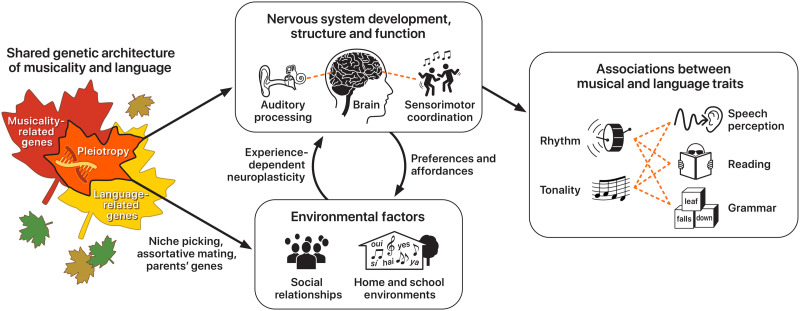
The MAPLE framework. Genetic pleiotropy is illustrated by overlapping maple leaves (left) signifying shared polygenic architecture influencing musicality and language traits. Shared genes influencing musicality and language are thought to exert influence on cascading biological processing including development, structure, and functioning of the brain, and nervous system functions relevant for musical and language traits such as auditory processing, and sensorimotor coordination (center top). Alongside genetic influences on these neural endophenotypes, polygenic influences on musicality and language are also thought to influence key environmental factors such as social relationships, and home musical and language environments (center bottom). Thus, genetic pleiotropy (left) is thought to be a root biological mechanism underlying the observable phenotypic associations between musical and language traits widespread in the literature (right). The MAPLE framework’s predictions can be tested by examining (a) polygenic architecture underlying musicality and language traits (left), (b) polygenic architecture and heritability of relevant neural endophenotypes, and of home and school musical and linguistic environments (center top and bottom, respectively), and (c) phenotypic variation in musical and language traits in broad populations, and cross-trait associations (right). Arrows illustrate the directions of influence between genes, neural endophenotypes, environment, and behavior.

Based on the hypothesized relationship between musicality and language abilities across the lifespan (Figure 1), we propose the Musical Abilities, Pleiotropy, Language, and Environment (MAPLE) framework for clarifying biological mechanisms of this lifespan view of musicality-language relationships (Figure 2). Briefly, the MAPLE framework proposes that observed phenotypic associations between musicality and language-related traits are partly driven by shared genetic architecture, and that these musicality-language links are further reinforced by **gene-environment interactions** and neuroplasticity.

Central to the MAPLE framework is the idea that genetic architectures underlying musicality and language traits partly overlap through the mechanism of genetic **pleiotropy**. Since both musical and language traits are complex and polygenic (i.e., affected by multiple genes in concert, rather than individual genes), we henceforth refer to this overlap in polygenic architectures as polygenic pleiotropy. An emphasis on shared genetic contributions allows us to leverage the known variability and heritability of musical skills (Niarchou et al., 2022; Ullén et al., 2014) and speech-language skills (Deriziotis & Fisher, 2017; Evans et al., 2015; Graham & Fisher, 2013). Further, since there is evidence of neural overlap in music and language processing (Atherton et al., 2018; Patel, 2012; Perani, 2012; Peretz et al., 2015), genetic variants that influence neurobiological structure, function, and development are also expected to be statistically overrepresented (relative to genes influencing other non-nervous system function) among loci common to musical and language traits (see Fisher & Vernes, 2015, for a detailed discussion of how genes affect neuronal circuits relevant for language). The MAPLE framework therefore emphasizes the importance of neural endophenotypes that mediate the relationship between genetic architecture and music/language phenotypes.

Alongside their influence on musical and language *abilities*, genes also exert influence on music and language-related *environments*—including environments shaped by oneself through increased or decreased *engagement*. For example, home environments during early development partly reflect the genetic predispositions of parents, through **niche-picking**, as in when musically talented parents choose to pursue musical training for their children. Since children inherit their parents’ genetic makeup, they too may niche-pick certain experiences compatible with their inherited aptitude and preferences (Hart et al., 2021). The effects of parents’ genes, and the environments that are created as a result, are amplified when children inherit stronger genetic predispositions for music and/or language traits through the process of **assortative mating** (Burley, 1983), as in when individuals seek out mates with similar levels of musicality. In addition to the genetic influences on the environment, biological/neurocognitive **affordances** can also directly affect the experiences sought and enjoyed by individuals.

The MAPLE framework is consistent with existing evidence of musical experience-dependent neuroplasticity across the lifespan (Merrett et al., 2013; Münte et al., 2002; Schlaug et al., 2009). Of interest here, musical training is thought to be strongly associated with higher performance on measures of auditory skills (Kraus & Chandrasekaran, 2010), language skills (Gordon, Fehd, & McCandliss, 2015; Tierney & Kraus, 2013; White et al., 2013), and executive functioning (George & Coch, 2011; Moreno et al., 2011). Moreover, the framework allows us to account for genetic influences on the brain and nervous system functions, to explain how biological mechanisms (e.g., gene regulation; neuroplasticity) interact to give rise to covariance between musical and language abilities across the lifespan. The MAPLE framework complements and extends existing frameworks linking musical and speech-language abilities, such as the atypical rhythm risk hypothesis (Ladányi et al., 2020). While the atypical rhythm risk hypothesis is primarily concerned with genetic and neural links between atypical rhythm and risk for speech-language disorders, the MAPLE framework considers individual differences in typical speech-language outcomes at the population-level, highlighting the role of musical abilities and related biology. The hypotheses and predictions of the MAPLE framework are compatible with those laid out by the atypical rhythm risk hypothesis.

The approach described here is akin to current frameworks in the field of psychiatric genetics. Researchers have highlighted shared genetic influences that underlie comorbidities among disorders both within disorders of a similar type, such as genetic overlap among mood and anxiety disorders; and across broad categories of psychopathology, such as genetic overlap across mood/anxiety disorders and thought disorders (Kotov et al., 2017; Selzam et al., 2018). While these conditions often present as clinically distinct, by interrogating common genetic influences that confer risk for psychopathology, researchers have been able to discover shared characteristics of traditionally separate conditions. For example, overlapping genetic architecture between psychiatric disorders can exert influences on underlying neurodevelopmental mechanisms that transcend diagnostic categories (Lee et al., 2019). Further, unraveling genetic pleiotropy between psychiatric disorders can improve nosology through characterizing relevant domains (e.g., sensorimotor), constructs (e.g., action planning), and units of analysis (e.g., genes and neural circuitry) underlying different disorders, as outlined by the research domains criteria (RDoC) framework (Cuthbert, 2014). Neurogenetic discoveries such as these can lead to identifying novel neurobiological risk or resilience factors for various psychiatric conditions. Similarly, this approach can be applied to musicality and language traits to help us better characterize the neurobiological factors that give rise to individual differences in both sets of skills and abilities, and covariation between them.

## INTEGRATING GENETICS APPROACHES INTO MUSICALITY-LANGUAGE RESEARCH

Genetic designs can inform the associations between musicality and language traits in several ways. First, the classic twin design can decompose phenotypic correlations into genetic, shared environment, and nonshared environmental correlations. The presence of a strong genetic correlation would indicate that a common set of genetic influences gives rise to individual differences in both sets of traits. In contrast, the presence of shared or nonshared environmental correlations could indicate that associations are driven by environmental exposures, potentially including causal relationships, though there are other ways of testing for causal associations in the context of a twin model (Heath et al., 1993). Twin and family studies can also test for the presence of gene-environment correlations and gene-by-environment interactions, which appear highly relevant for musical traits (Hambrick & Tucker-Drob, 2014; Wesseldijk et al., 2019). These studies have demonstrated that individual differences in music achievement are more pronounced in those who engage in practice or had musically enriched childhood environments. Similar work could examine whether individual differences in language abilities are more pronounced for individuals with more musical experiences or stronger musical abilities (for guidelines on statistical models that can be tested, see Purcell, 2002; van der Sluis et al., 2012).

One example of the utilization of twin and family studies to examine the associations between musicality and language comes from Gustavson et al. (2021), who find that self-reported musical instrument engagement at age 12 predicts verbal ability at age 16, controlling for IQ, and that this relationship is likely explained by shared genetic influences. Further, Wesseldijk et al. (2021) report recent findings in twins that demonstrate that phenotypic associations between musical aptitude and vocabulary skills are partially attributable to shared family influences. These methods allow for an estimate of the influences of genetics on musical and language skills in the populations being studied. Further, while the effects of music environments have traditionally been explained via models of neuroplasticity, behavioral genetics findings allow us to reimagine what musical environments can tell us.

Beyond the classic twin design, large-scale genomic data can also be useful to uncover musicality-language links and inform us about the nature of their shared biology. Large-scale **genome-wide association studies** (GWAS) for example, involve scanning markers across the complete genome of many people to find common genetic variations associated with a particular trait. Consistent with the polygenic nature of complex human traits, each of these common variations is likely to have only a small effect size, necessitating large sample sizes (thousands of participants) to reliably detect associations. By identifying these genetic associations with the trait of interest, we can better understand the genetic architecture of the trait. Further, **polygenic scores** (PGSs; Krapohl et al., 2018) applied to individuals’ genomic data, with weights at specific alleles derived from the results of large-scale GWAS, can be used to develop better strategies for detecting which individuals might have genetic predispositions that explain variance in another phenotype (e.g., risk for dyslexia or DLD based on relatively lower polygenic scores for a language-related trait).

While GWAS efforts for cognitive traits have largely focused on general cognitive ability (Davies et al., 2018; Savage et al., 2018), such studies have more recently begun to shed light on the molecular bases of speech, language, and reading (Eising et al., 2022; Graham & Fisher, 2013; see Deriziotis & Fisher, 2017, for extended discussion of language GWASs), including in the context of developmental **dyslexia** (Doust et al., 2022; Gialluisi et al., 2021). The first large GWAS of a musical trait, beat synchronization (Niarchou et al., 2022), has demonstrated highly polygenic architecture of this rhythm-related trait. As GWAS of musical and language-related traits become more widely available, genetic correlations (Bulik-Sullivan et al., 2015) between these traits can be estimated even when the samples used for GWAS do not overlap.

Relatedly, we can utilize the result of extensive mapping of regions of the human genome to biological functions, included those involved in neural development and other processes. For example, Niarchou et al. (2022) found that the genetic architecture of beat synchronization is **enriched** for brain specific regulatory regions of the genome, in both adult and fetal brain tissues. In this way, statistically integrating neurogenomic data will allow us to map the genetic architecture of musicality and language traits to neurodevelopmental and neurofunctional endophenotypes. Application of such methods and the subsequent findings would represent a major advance towards the goal of testing the MAPLE framework’s predictions about shared genetic architecture between musicality and language (see the section Predictions Derived from the Maple Framework).

Moreover, researchers can draw on the potential of genomic data by using available **polygenic scores** for phenotypes of interest to examine associations with traits in target samples for whom genotyping is also available (provided the samples are of adequate size for capturing polygenic signal; usually comprising tens of thousands to millions of individuals for *computing* polygenic scores from discovery GWASs, and hundreds to thousands of individuals for *applying* polygenic scores to target samples). For example, given cohorts with language measures, we can examine to what extent individual differences in genetic predispositions for musical rhythm ability (e.g., using polygenic scores computed from a large discovery GWAS of musical rhythm) are predictive of performance on reading-related tasks (in a more modest target sample). This approach can be incorporated into any of the behavioral/neural designs commonly utilized in the literature on musicality-language links. Polygenic scores can also be examined as moderators, for example to test whether individuals with high genetic risk for language disorders show stronger language outcomes when exposed to musical experiences at an early age. Given the broad and intertwined (pleiotropic) influence of genes on (neuro)biology, genomic approaches can reveal shared biological mechanisms of musicality and language traits that converge across various constructs, tasks, and stimuli common in the literature (detailed in the Literature Review section).

Computational genomic approaches can also be used to investigate the evolutionary basis of complex traits along lineages that led to present-day humans as well as comparatively across species. For example, Gordon et al. (2021) examined overlapping genetic architecture between beat synchronization traits in humans, and complex vocal learning traits in songbirds. That study provided genetic evidence for Patel’s (2021) hypothesis of convergent evolution between songbird vocal learning and human beat perception and synchronization, which posits that musical rhythm processing piggybacks on neural circuitry evolved for complex vocal learning. Similarly, computational approaches can be used to shed light on shared or separate evolutionary histories of traits of interest (Colbran et al., 2019; Tilot et al., 2021). As additional sets of GWAS data become publicly available, the information can be used to validate and extend results from phenotypic factor analyses, through genomic structural equation modelling techniques that make it possible to analyze the joint genetic architecture of complex traits (e.g., Gustavson et al., 2020) as well as the genetic loci implicated in divergence between traits (Grotzinger et al., 2019). These advances can more directly inform our understanding of polygenic pleiotropy between musical and language traits proposed by the MAPLE framework.

Future GWAS efforts should target some of the behavioral and neural endophenotypes that have been highlighted by prior studies of musicality-language links, as reviewed later in this article. Given efforts currently underway to map the genetic architecture of neural traits (e.g., work by the ENIGMA consortium: Smit et al., 2021) there is a timely opportunity to leverage existing resources to further our understanding of musicality-language links. Large-scale data sets can now be creatively leveraged to map genetic variants to neural correlates of musicality and language traits (e.g., Mekki et al., 2022, recently mapped the genetic architecture of resting-state functional connectivity in brain regions classically associated with language function). To identify neural endophenotypes as proposed by the MAPLE framework, future research can systematically draw on existing neural data that is linked with genetic and behavioral data, for example in biobank initiatives such as the UK Biobank (Sudlow et al., 2015). While ideally, musical and language abilities will eventually be deeply phenotyped in study participants in large-scale population health studies (e.g., All of Us: Kaiser, 2016), in the meantime we can also leverage neural maps based on meta-analyses of music and language processing or abilities (e.g., Kasdan, Burgess, et al., 2022), to guide neurogenetic investigations.

Recent advances in data-driven approaches such as data mining algorithms and machine learning models have the potential to further accelerate progress in this research area, by automating the identification of cases with particular symptomatology in electronic heath records (e.g., automated phenotyping tool for DLD cases, APT-DLD: Walters et al., 2020; phenome risk classifier for stuttering: Pruett et al., 2021), the extraction of neural features from magnetic resonance imaging (MRI) data (e.g., toolbox for the automatic segmentation of Heschl’s gyrus, TASH toolbox: Dalboni da Rocha et al., 2020), the extraction of features in the genetic architecture of a trait (e.g., GWAS loci prioritization: Nicholls et al., 2020), and the integration of neuroimaging and genomic data to predict phenotypic outcomes (e.g., Shen & Thompson, 2020).

Last, longitudinal studies investigating how early musical skills may predict aspects of language development can additionally incorporate genetic data (i.e., via polygenic models of musical abilities and engagement, and of brain function) to begin to chart gene-environment interactions during development, accounting for predisposition and neural plasticity (Zuk & Gaab, 2018).

## PREDICTIONS DERIVED FROM THE MAPLE FRAMEWORK

The MAPLE framework can be used to guide many kinds of research questions, which can be answered by utilizing genetic and genomic approaches (Box 1, numbered). For each research question, specific predictions can be made about anticipated outcomes (Box 1, bulleted).

**Box 1.** Research questions and predictions about musicality-language links.

**1)**  **Do musicality and language share genetic architecture? If so, which musical traits are genetically associated with which language traits?**
  - Analyses performed on the results of well-powered music and language GWAS will show significant genetic correlations between musical and language traits that are known to be phenotypically correlated (e.g., using linkage disequilibrium score (LDSC) regression methods: Bulik-Sullivan et al., 2015) across various constructs and stimulus types.
**2)**  **What can be predicted about phenotypic variability in language traits based on genetic variation associated with musicality, and vice versa? How are these relationships mediated by genetic predictor models of brain activity and structure?**
  - Polygenic scores (PGSs) for musical traits derived from one sample will predict behavioral speech/language/reading phenotypes in an independent sample, and vice versa.
  - These PGS associations will be mediated by PGSs for neural endophenotypes already shown to underlie the relation between specific musicality and language traits.
**3)**  **Which neurobiological functions are the genes associated with musicality and language traits enriched for? Do they implicate systems already known to be involved in musical and language abilities?**
  - Enrichment analyses (e.g., Subramanian et al., 2005) performed on the results of well-powered GWAS of musicality phenotypes will show that the genetic architecture of musicality is enriched for genes involved in neural endophenotypes of language/communication. These neural endophenotypes may, for example, include the following:
    (1)  Hierarchical processing, music and language processing, or auditory processing: superior temporal gyrus, Heschl’s gyrus volume; inferior frontal gyrus (Broca’s area).
    (2)  Temporal brain dynamics: motor system functionality (e.g., for circuits involving cerebellum, and/or basal ganglia); resting state neural oscillations; oscillatory mechanisms of music or language perception.
    (3)  General cognitive abilities across the lifespan: white matter connectivity; surface area and thickness of cortical regions; subcortical volumes; cerebral volume (see Bethlehem et al., 2022).
  - Similarly, enrichment analyses performed on the results of well-powered language-related GWAS will show that the genetic architecture of language-related traits is enriched for genes associated with neural endophenotypes involved in music perception and cognition.
  - Functional genomics techniques (e.g., imputed gene expression analysis; Gamazon et al., 2015, 2019) will show that genes associated with musicality traits in well-powered GWAS are expressed in neurobiological and neurodevelopmental processes known to underly language traits.
**4)**  **How do neural endophenotypes support co-varying musical and language traits?**
  - PGSs for musical traits derived from well-powered samples will mediate relationships between individual variation in neuroimaging correlates (e.g., EEG; MEG; DTI) and language phenotypes in an independent sample, and vice versa.
  - Multivariate analyses (e.g., Grotzinger et al., 2019) will uncover the shared genetic architecture between music, language, and relevant neural endophenotypes.
**5)**  **When in development are genetic predispositions most sensitive to environmental factors in influencing outcomes for musical and language abilities? When are genes associated with musicality-language links expressed in (neuro)biological systems of interest?**
  - Longitudinal or cross-sectional developmental studies will show enrichment for genes expressed or regulated during specific stages of neurobiological development (e.g., in fetal, neonatal, and infancy) in genes common to musical and language traits.
  - Longitudinal or cross-sectional developmental and lifespan studies will show that genetic risk/resilience for musical traits will differentially predict speech-language trajectories across early development (e.g., increase in performance across childhood and adolescence) and aging (e.g., maintenance and decline across middle age and old age).
**6)**  **Who is most at-risk for speech and language difficulties? How can we better identify these individuals?**
  - Individuals with higher polygenic risk scores for music-related impairments will be more likely to have clinical speech-language difficulties.

### Testing the MAPLE Framework: An Agenda for Musicality-Language Research

Strategic directions for testing the MAPLE framework could be supported by the following approaches:

1)  Integrating genetic approaches into music and language science:
  - Add musicality variables to genetically informative samples across a range of cognitive, neurobiological, and speech-language phenotypes.
  - Investigate pleiotropy with cross-trait methods such as genetic correlations and polygenic score analysis. Focus on areas where there are strong phenotypic links, such as those found in the literature synthesis here, to guide investigations of polygenic pleiotropy.
  - Explore neural endophenotypes in relation to genes and behavior in large samples and across traits measured in separate samples, using state-of-the-art methods and by leveraging big data.
2)  Integrating music science with behavioral and neural approaches to language:
  - Include musical abilities in models examining individual differences in language skills, to account for covariance.
  - Capture rich variation in language experiences, backgrounds, and engagement alongside variation in musical traits, going beyond language outcomes and skills.
3)  Considering genetic explanations alongside transfer effects:
  - Consider hypotheses that compete with, or are complementary to, neuroplasticity-based frameworks, when explaining behavioral outcomes studied using correlational designs. For example, test genetic and gene-environment interaction-based hypotheses, alongside neuroplasticity-based frameworks, when two behavioral traits such as music engagement and language abilities are related.

### How Can the MAPLE Framework Advance the Field?

1)  ***Genetic architecture***: The MAPLE framework can guide our understanding of the genetic basis of the human capacity of music and language, their genetic associations, their genetic influences on mediating biological and neural endophenotypes, and their shared evolutionary histories (see Box 1 and Box 2 for further discussion).
2)  ***Individual differences***: The MAPLE framework can help clarify covarying individual differences in musicality and language, including interactions between genetic, neurobiological, cognitive, and environmental factors influencing these links. The framework can also help better characterize known dissociations between music and language processing (Chen et al., 2021; Clos et al., 2013). These dissociations can occur in the presence of shared genetic architecture but non-shared neural architecture. Further, the framework allows for temporary state-level dissociations between musicality and language, while accounting for stable trait-level associations (see Box 2 for further discussion).
3)  ***Clinical efforts for speech-language disorders***: Given emerging evidence that individuals with musical impairments are often at higher risk for language-related disorders (Ladányi et al., 2020), the testable genetic predictions of the MAPLE framework can help us better understand biological mechanisms of risk profiles for speech-language disorders, and possibly identify biomarkers to aid early identification and timely intervention. For example, accounting for polygenic risk scores for musical impairments (e.g., rhythm perception) could potentially serve as a powerful transdiagnostic approach cutting across clinically distinct speech-language disorders (Lense et al., 2021; Sauer-Zavala et al., 2017). Further, testing the MAPLE framework will help validate neurobiological and behavioral dimensions that can bridge current developmental disorder categories, as called for by the research domains and constructs (RDoC) framework (Cuthbert, 2014). This in turn may offer more nuanced or alternative ways of diagnosing and classifying disorders (e.g., the DSM and ICD manuals), and improved precision treatment approaches based on specific (neuro)genomic risk profiles.
4)  ***Cognitive science debates***: The grounding of the MAPLE framework in polygenic pleiotropy provides new directions for intervening productively in long-standing cognitive science debates concerning the domain-specificity or domain-generality of aspects of music and language (Besson & Schön, 2012; Brandt et al., 2012; Lagrois et al., 2019; Morrill et al., 2015; Swaminathan et al., 2018), and about cognitive and neural resource sharing between music and language processing (e.g., Abrams et al., 2011; Patel, 2012, 2021; Peretz et al., 2015). For example, musical rhythm perception and reading-related processing may recruit partially distinct neural networks and brain regions, but these traits may still be influenced by shared genetic architecture, which can be clarified using genomic approaches (see Box 2 for further discussion).
5)  ***Evolutionary theories***: By focusing on shared genetic architecture, the MAPLE framework is consistent with theories that posit common evolutionary and biological roots for the musical and language abilities of our species (Brown, 2017; Mehr et al., 2021; Morley, 2013; Mithen, 2005; Patel, 2012, 2021). See Box 2 for further discussion.

**Box 2.** Disentangling genetic and neural architecture of musical and language functions in the brain using the MAPLE framework. While the MAPLE framework is primarily focused on associations between musicality and language traits, there is some evidence from functional neuroimaging of dissociations between brain networks recruited for language and musical functions (Chen et al., 2021; Norman-Haignere et al., 2022; Peretz et al., 2015). We argue that the MAPLE framework cannot only co-exist with findings of neural dissociations between musical and language processing, but can also help further characterize them in the following ways.

**1)**  **The MAPLE framework is consistent with other frameworks linking music and language, which predict the dual phenomena of neural *sharing* and neural *specialization* underlying musical and linguistic function (e.g., Patel, 2021).**
  There are many possible ways in which the genetic architecture of complex traits could manifest in cross-trait phenotypic associations, but the central tenet of the MAPLE framework is that there is shared biology between musicality and language traits. Nevertheless, we do also expect some unique/non-shared variance in individual differences in these traits, as well as in their underlying neural mechanisms. That is, phenotypic correlations between musicality and language could arise from shared genetic alleles at specific locations on the genome (i.e., loci) that influence *separate* components of neural circuitry, alongside shared components.
  *Consider the following example regarding musical rhythm and reading traits*:
  As reviewed in the Musicality, Reading Readiness, and Literacy section, there are robust and well-replicated associations between rhythm and reading skills, despite the signal characteristics of musical rhythms and printed word being quite distinct. Moreover, current knowledge about the networks of brain areas activated during reading (Martin et al., 2015) and musical rhythm tasks (Kasdan, Burgess, et al., 2022) suggests that they are minimally overlapping (although more research is needed to directly disentangle potential neural overlap).
  Yet, it is well-established that reading skills are supported by phonological learning (Snowling, 2001), which in turn has many parallels to complex vocal learning, the subject of Patel’s (2021) revised vocal learning hypothesis. Recent genomic results demonstrate that songbird vocal learning exhibits shared genetic architecture with human beat synchronization (Gordon et al., 2021), thus suggesting convergent evolution evidence in support of the revised vocal learning hypothesis. Patel describes possible delineation within language circuitry that may have co-evolved with rhythm, specifically predicting the dorsal stream of the language network (more so than the ventral stream) to be tuned to complex vocal learning and phonological processing, and to share circuitry with beat perception and synchronization. The dorsal stream may be a particularly interesting candidate area to explore the relationship between shared neural and genetic architecture of rhythm and phonological processing (Patel, personal communication).
  Phenotypic correlations between musical rhythm and reading could be thus explained by the MAPLE framework in multiple ways:
  - Shared genetic architecture (i.e., the same sets of alleles at hypothesized common genetic loci between rhythm and reading traits) acting upon shared neural circuitry (e.g., dorsal stream areas).
  - Shared genetic architecture acting on separate components of the neural circuitries of rhythm and phonological processing (e.g., neuroimaging studies show separate neural activation patterns when processing rhythmic vs. phonological stimuli, while genetic correlations are still found between rhythm and phonology/reading-related traits due to pleiotropy).
  - Separate genetic architecture acting on common components of these neural circuitries (e.g., genes at different loci that influence brain structure and function in some overlapping areas/processes between rhythm and reading).
**2)**  **The MAPLE framework can account for the biological basis of associations between stable individual differences in musicality and language *traits*, even in the presence of biological dissociations between musicality and linguistic *states*.**
  Here, it is important to clarify conceptual distinctions between variation in *traits* and variation in *states*. Trait-dependent variance captures variation across individuals relative to group means, whereas state-dependent variance captures variation over repeated observations of the same individual over time, allowing for changes in environmental, or developmental factors (Sanchez-Alonso & Aslin, 2020). Genomic variation between people is known to contribute to constitutive individual differences in musicality and language traits (e.g., rhythm discrimination abilities or morphosyntactic skills). On the other hand, experimental tasks that are designed to observe group-level differences in neural processing, across repeated observations (e.g., syntactic congruity vs. syntactic violation conditions), instead target the state-level, and are often not well suited for capturing stable individual differences in traits (see Hedge et al., 2018, for an extended discussion about trade-offs between stable individual differences metrics and reliable experimental effects). Thus, individual or group differences found in brain responses to musical or linguistic states do not necessarily need to converge with the central focus on the current work: stable individual differences in musicality and language traits. Predictions of the MAPLE framework about phenotypic and genetic overlap between individual differences in musical and language abilities are therefore consistent with evidence for neural dissociations between musical and language states, such as:
  - Chen et al.’s (2021) finding that language-specific brain regions appear to have no sensitivity to structural musical violations, and that individuals with aphasia who showed grammatical processing impairments had intact tonal music perception.
  - Peretz et al.’s (2015) finding that there appears to be a great deal of neural specialization *and* neural sharing between musical and speech processing, and their crucial reminder that neural overlap (as defined by neuroimaging studies) does not in itself mean neural resource sharing. They argue that it is possible that neural circuits appear overlapping because they are intermingled or adjacent.
  - Norman-Haignere et al.’s (2022) finding of a neural population that responds specifically to music with sung melodies, but that does not respond to other musical stimuli or speech stimuli.

**Future Directions:** As neurogenetic advances are made to map genome-wide influences on more nuanced functional networks, neural populations, and microstructures, the MAPLE framework will allow us to characterize the genetic influences on neural specialization (i.e., non-shared elements), as well as neural sharing, between musicality and language traits.

## SUPPORTING EVIDENCE FOR THE MAPLE FRAMEWORK

The remaining sections of this article provide an overview of the broader field of research examining links between musicality and language, and a detailed (non-exhaustive) thematic review of patterns found specifically within the individual differences literature. Studies discussed below provide supporting evidence for the MAPLE framework, and point to relevant phenotypes (behavioral evidence of correlations), and potential neural endophenotypes (neural correlates of musicality-language associations), that can become a focus of future genetics investigations.

### State of the Musicality-Language Field

**Figure 3.**
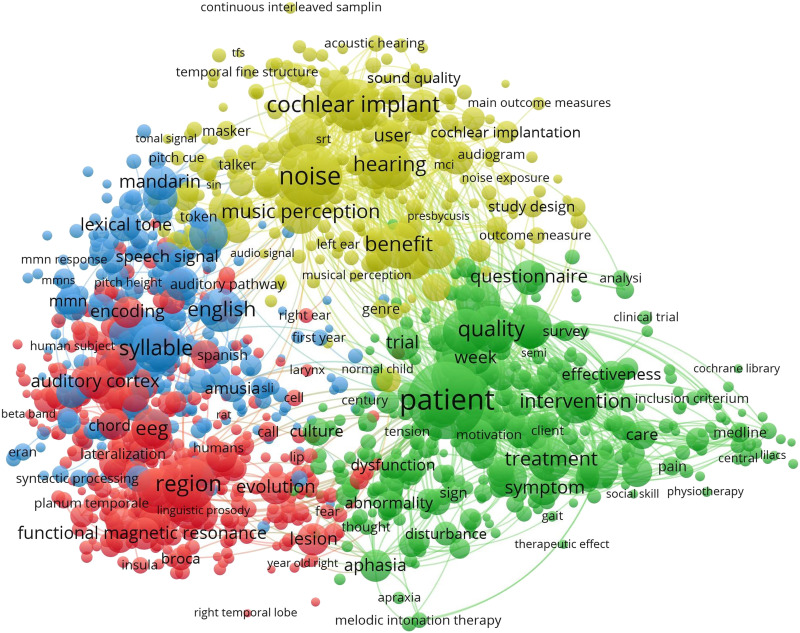
Bibliometric mapping based on co-occurring music and language terms. Figure shows the major clusters of research areas emerging when 60% of the most relevant terms shared between published articles are shown. Search terms included common terms associated with music and language science.

We conducted bibliometric mapping based on connected terms in the literature to understand where *individual differences* approaches (the focus of the present work) are situated in the larger research area of music and language, and relative to *musical training* approaches (see the Supporting Information for further details on bibliometric mapping). Briefly, representative terms related to music and language were searched in PubMed and visualized using VOSviewer (van Eck & Waltman, 2010). The analysis showed four major emerging clusters in the literature, based on connections between terms: (1) Music and speech perception in the context of hearing, hearing loss, and cochlear implant users (Figure 3, yellow); (2) Music and language processing in the context of tonal language backgrounds and second language learning, with implications for reading-related disorders such as dyslexia (Figure 3, blue); (3) Neuroimaging of music and language, in healthy brains as well as contexts of brain injury or lesions (Figure 3, red); (4) Music in the context of clinical interventions and related intervention outcomes, e.g., for treatment of speech-language pathology (Figure 3, green).

**Figure 4.**
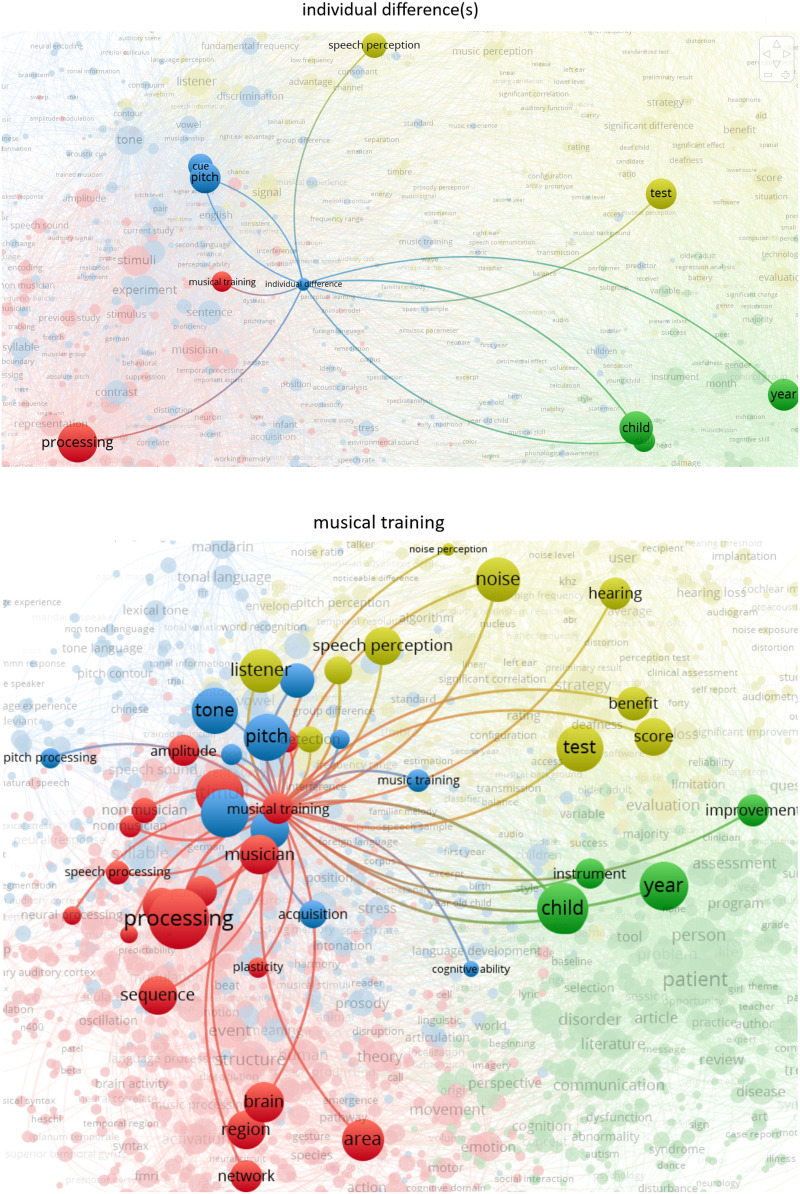
Figure mapping “individual difference” and “musical training” within bibliographic clusters. When the term “individual difference” occurred in publications, it was most commonly accompanied by the terms “speech perception,” “test,” “child,” “skill,” “year,” “processing,” while “musical training” was linked to a much wider array of terms representing its more widespread use in the music and language science literature. Plurals in the figure are encompassed by singular forms (e.g., “individual difference” also comprises “individual differences”).

We queried the term “individual differences” and found it had 84 occurrences, which indicates that this area is attracting attention. In contrast, the terms “formal musical training,” “musical training,” and “music training” together showed 360 hits, accounting for a much larger portion of the academic interest in the links between music and language. Further, “musical training” was linked to a wider variety of terms within each of the four clusters relative to “individual differences” (Figure 4; see Figure S1 for additional details). Taken together, these analyses provide a bibliometric overview of musicality-language studies and highlight “individual differences” as an emerging area.

### Literature Review: Associations Between Individual Differences in Musical and Language Abilities

#### Individual differences literature in a nutshell

**Figure 5.**
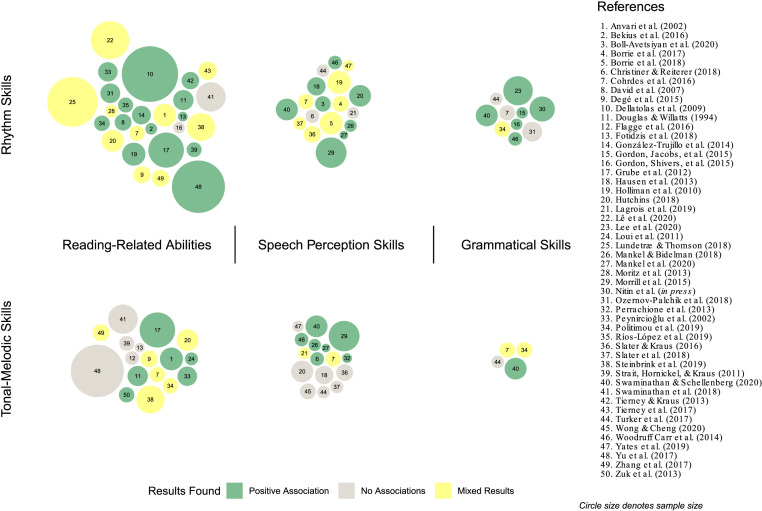
Studies examining within-sample associations between musicality and language traits. Each bubble corresponds to a single study, and studies are grouped by which broad language-related construct(s) they examined (e.g., speech perception, grammatical skills, reading-related abilities, or multiple) on the horizontal axis, and by which musicality construct(s) they examined (e.g., tonal-melodic or rhythm skills) on the vertical axis. Studies spanning multiple musicality or language traits are represented by multiple bubbles as relevant.

To examine phenotypic associations between musicality and language-related traits, we synthesized studies reporting correlations between musical and language abilities. Specifically, we focused on associations found in the form of significant positive correlations between at least one musicality-related and one language-related phenotype. Studies can be broadly classified as examining associations between musical rhythm or tonal-melodic skills, and speech, grammar, or reading-related traits. (See Supporting Information for details on the literature search terms that were used to inform the framework. Note that this literature synthesis is theoretically driven and not meant to be exhaustive or to take the place of a systematic review or meta-analysis.) The literature reviewed here represents a variety of samples including adults, children, and infants; individuals with or at-risk for language-related disorders; and second language (L2) learners, foreign language (FL) learners, or individuals with tonal language backgrounds (e.g., Cantonese). Figure 5 provides an overview of studies that inform our framework, demonstrating accumulating and converging evidence that musicality covaries with multiple language-related traits across a broad population. The literature provides extensive evidence for positive correlations between individual differences in musical and language abilities, spanning both rhythm and tonal-melodic measures, and speech-language and reading measures. Figure 5 also provides a snapshot of mixed results and areas of relative sparsity to guide future research efforts.

#### Musicality and speech perception

##### Behavioral evidence: Musical abilities are correlated with speech task performance.

In the context of spoken languages, successful speech perception is fundamental to language acquisition and communication. Speech perception involves many complex processes including segmentation of auditory speech streams; parsing the embedded prosodic and rhythmic patterns of speech to perceive tone, context, emotion, and pragmatic information during discourse; and being able to hear speech in non-ideal or noisy talker conditions. Beyond typical development, speech perception deficits are associated with speech-language and reading disorders such as dyslexia (Goswami, 2011). Many studies have previously found that musicians show greater perceptual sensitivity to speech (Bidelman & Alain, 2015; Magne et al., 2006; Marie et al., 2011), compared to non-musicians. Here, we synthesize findings from the studies shown in Table 3, showing associations between individual differences in musicality and speech perception phenotypes.

**Table 3.** Studies reporting associations between musicality and speech perception.

**Study**	***N***	**Age**	**Rhythm construct**	**Tonal-melodic construct**	**Speech perception construct**
Boll-Avetisyan et al. (2020)	46 (23 dyslexic)	17–35	Rhythm discrimination		Speech rhythm sensitivity
Borrie et al. (2017)	50	18–29	Rhythm discrimination		Speech rhythm sensitivity
					Speech learning
Borrie et al. (2018)	98	22–62	Rhythm discrimination	Melody discrimination	Speech rhythm sensitivity
Christiner and Reiterer (2018)	35	5–6	Rhythm discrimination	Melody discrimination	Speech imitation
Cohrdes et al. (2016)	44	5–7	Rhythm reproduction	Melody reproduction	Speech pitch perception
			Beat synchronization	Harmonic discrimination	
Hausen et al. (2013)	64	19–60	Beat perception	Melody discrimination	Speech prosody perception
				Harmonic perception	
				Pitch perception	
Holliman et al. (2010)	102	5–7	Rhythm reproduction		Speech rhythm sensitivity
			Rhythm discrimination		
Hutchins (2018)	90	3–6	Beat synchronization	Singing error	Speech-in-noise perception
Mankel, Barber, and Bidelman (2020)	14	23–27	Beat discrimination	Melody discrimination	Speech processing
			Tempo discrimination	Harmonic discrimination	
Mankel and Bidelman (2018)	28	19–25	Beat discrimination	Melody discrimination	Speech-in-noise perception
			Tempo discrimination	Harmonic discrimination	
Morrill et al. (2015)	179	18–49, *M* = 19.9	Rhythm discrimination	Melody discrimination	Speech prosody perception
			Metrical perception	Musical memory	
Perrachione et al. (2013)	18	18–30		Melody discrimination	Speech pitch perception
Slater and Kraus (2016)	54 males	18–35	Rhythm discrimination	Melody discrimination	Speech-in-noise perception
Slater et al. (2018)	31 (17 drummers)	*M* = 24.7	Beat synchronization	Pitch discrimination	Speech-in-noise perception
Swaminathan and Schellenberg (2020)	91	6–9	Rhythm discrimination	Melody discrimination	Speech perception
				Musical memory (melodic)	
Woodruff Carr et al. (2014)	35	3–4	Beat synchronization	Melody discrimination	Speech encoding
			Rhythm discrimination		
Yates et al. (2019)	24	19–40	Beat synchronization	Melody discrimination	Speech-in-noise perception
			Rhythm discrimination		

*Note*. Rows correspond to individual studies, and columns correspond to study *N*s, age ranges in years, specifics of musical rhythm and tonal-melodic constructs measured, and speech constructs measured. When additional information is necessary about the *N*, specific descriptions are given in parentheses. When age range is not available, or not representative of the sample mean, the mean is provided in addition to the age range. Only musical constructs that were reported in relation to speech perception constructs are noted.

Given that speech prosody partially captures the rhythms embedded in speech, it is perhaps unsurprising that musical rhythm sensitivity is emerging as a predictor of speech rhythm perception (Hausen et al., 2013; Morrill et al., 2015). Beyond rhythm, individual differences in musical pitch contour discrimination, and parsing pitch contours in speech, are also associated (Cohrdes et al., 2016; Perrachione et al., 2013). This suggests that underlying neural and genetic architecture may be shared between musical and speech processing. Importantly, these associations reinforce the value of studying tonal-melodic perceptual abilities in the context of *non-tonal* languages, alongside the more common study of *lexical* tone (illustrated in Figure 3), discussed further in the Musicality, Second Language Learning, and Tonal Language Development section.

Musical rhythm skills, specifically, appear to be associated with speech discrimination (Swaminathan & Schellenberg, 2020) and speech perception in noise (SPIN: Slater et al., 2018; Slater & Kraus, 2016; Yates et al., 2019), in both children and adults. Yates et al. (2019) also report positive correlations between musical experiences and training (both formal and informal) and SPIN. These results leave open the possibility that SPIN abilities may be shaped by musical experience, while interacting with (potentially genetically driven) individual differences in musical and speech listening skills (e.g., Bidelman & Alain, 2015). For example, individuals with higher musical aptitude may gravitate towards music training (i.e., gene-environment correlation). The heritability of speech perception task performance is largely unknown, and information about this would be beneficial for genetic efforts towards understanding music-speech links. At present, there is evidence that both speech articulation (Table 1) and *non-speech* auditory perception in noise (Brewer et al., 2016) are moderately heritable.

Alongside perceptual abilities, rhythm production and imitation are also associated with speech encoding and SPIN (Slater et al., 2018; Woodruff Carr et al., 2014). For example, the ability to accurately reproduce drumming sequences, drum in time to the beat of a song, and to a metronome, were all correlated with SPIN in adults (Slater et al., 2018), pointing to the involvement of sensorimotor mechanisms. In the Slater et al. (2018) study, while beat synchronization was associated with SPIN in both non-musicians and percussionists, reproducing or imitating rhythmic patterns was only associated with SPIN in non-musicians, raising questions about interactions between genetic predispositions and experience-dependent neuroplasticity.

##### Neural correlates of associations between musicality and speech perception.

The behavioral associations discussed above are supported by neural studies of speech encoding in subcortical and cortical areas. For example, individuals with enhanced musical perception skills in general (Mankel & Bidelman, 2018), and rhythm skills more specifically (Tierney et al., 2017), show more efficient subcortical and cortical speech encoding mechanisms. In these studies, higher music aptitude was associated with enhanced neural responses involved in both fast (high-frequency) and slow (low-frequency) integration of auditory information over time.

Emerging neural evidence of music-speech links reflects the complex interplay between perceptual, cognitive, and motor traits involved in rhythmic abilities (see Cannon & Patel, 2021; Kotz et al., 2018). Tierney et al. (2017) showed that rhythmic abilities can be decomposed into at least two constructs—sequencing and synchronization—and that these factors are supported by differential brain networks involved in integrating auditory information. For example, cortical responses tracked sequencing (which correlated with language traits such as verbal memory and reading), whereas subcortical frequency-following responses tracked synchronization (which correlated with non-linguistic auditory task performance).

Other studies suggest that subcortical speech encoding and reading processing are intertwined, and are associated with musical rhythm (Strait et al., 2011) and categorization of speech sounds (Mankel et al., 2020). Based on evidence from preschoolers’ pre-reading skills, Woodruff Carr et al. (2014), and others have argued for an integrative biological framework in which speech encoding, “beat-keeping”/rhythm skills, and reading development are fundamentally linked.

Event-related potential (ERP) studies suggest that neural sensitivity to speech prosody is associated with musical rhythm abilities. For example, Magne et al. (2016) measured participants’ speech rhythm sensitivity using bi-syllabic words with the final word either matching or mismatching the stress pattern of the preceding words. Participants showed an increased fronto-central negative shift in ERPs in the stress mismatch versus match conditions, and the magnitude of this neural response predicted speech rhythm sensitivity. Another study (Fotidzis et al., 2018) found a similar negative shift when participants were visually presented with bi-syllabic words with a trochaic (strong-weak) stress pattern, preceded by weak-strong tones (i.e., mismatched stress patterns). The magnitude of the negative shift in ERPs was correlated with both rhythm aptitude and reading comprehension skills.

The above studies suggest some degree of shared neurobiology underlies musical and speech rhythm processing. Based on frameworks presented for the neural processing of beat and timing in auditory and speech stimuli (Kotz et al., 2018; Kotz & Schwartze, 2010), mechanisms underlying these correlations may involve previously identified subcortico-cortical networks involved in processing rhythmic aspects of music and speech (see Fiveash et al., 2021).

#### Musicality, reading readiness, and literacy

##### Behavioral evidence: Musical abilities are correlated with literacy-related skills.

By some accounts, almost two-thirds of twelfth graders in the United States do not possess fundamental skills required to master college-level reading material, and over 17% of adults have low English literacy (National Center for Education Statistics, 2019). Literacy is a crucial driver of socioeconomic (SES) outcomes: Individuals with low literacy skills are more likely to have fewer employment opportunities and earn lower wages (Decker & Decker, 1997), in addition to increased health risks (Baker et al., 1997). A host of reading-related skills are important building blocks for reading comprehension, including **decoding**, **fluency**, and **phonological awareness** skills. Table 4 provides further detail for studies examining associations between musicality and several reading-related phenotypes.

**Table 4.** Studies reporting associations between musicality and reading-related skills.

**Study**	***N***	**Age**	**Rhythm construct**	**Tonal-melodic construct**	**Reading-related construct**
Anvari et al. (2002)	100	4–5	Rhythm discrimination	Melody discrimination	Phonological awareness
			Rhythm reproduction	Harmony discrimination	Reading ability
				Harmony analysis	
Bekius et al. (2016)	26	20–40	Beat perception		Reading fluency
Cohrdes et al. (2016)	44	5–7	Rhythm reproduction	Melody reproduction	Phonological awareness
			Beat synchronization	Harmonic discrimination	
David et al. (2007)	53	*M* = 6.34	Beat synchronization		Phonological awareness
					Reading fluency
Degé et al. (2015)	55	*M* = 6.25	Rhythm discrimination	Melody discrimination	Phonological awareness
			Beat discrimination	Pitch discrimination	Nonword repetition
			Beat synchronization	Singing ability	
			Rhythm reproduction		
Dellatolas et al. (2009)	625	5–6	Rhythm reproduction		Phonological awareness
					Reading comprehension
Douglas and Willatts (1994)	78	7–9	Rhythm discrimination	Pitch discrimination	Word recognition/spelling
Flagge et al. (2016)	36	7–8		Pitch discrimination	Phonological awareness
González-Trujillo et al. (2014)	67	8–9	Rhythm reproduction		Reading fluency
Grube et al. (2012)	238	11	Rhythm discrimination	Pitch discrimination	Phonological awareness
				Timbre discrimination	
Holliman et al. (2010)	102	5–7	Rhythm reproduction		Reading ability
			Rhythm discrimination		Phonological awareness
Hutchins (2018)	90	3–6	Beat synchronization	Singing error	Phonological awareness
					Reading fluency
Lê et al. (2020)	278	*M* = 8.4	Beat synchronization		Phonological awareness
					Reading ability
Loui et al. (2011)	32	*M* = 7.6		Pitch discrimination	Phonological awareness
				Pitch production	
Lundetræ and Thomson (2018)	479	6	Beat synchronization		Phonological awareness
					Reading fluency
					Spelling
Moritz et al. (2013)	30	Starting at age 5, ending at age 7 (longitudinal)	Beat synchronization		Phonological awareness
			Rhythm reproduction		Reading ability
			Rhythm discrimination		
Ozernov-Palchik et al. (2018)	74	*M* = 5.8	Rhythm discrimination		Phonological awareness
					Reading fluency
Peynircioğlu et al. (2002) (1)	32	4–6	Rhythm reproduction	Melody production	Phonological awareness
Peynircioğlu et al. (2002) (2)	40	3–7	Rhythm reproduction	Melody production	Phonological awareness
Politimou et al. (2019)	46	3–5	Rhythm discrimination	Pitch discrimination	Phonological awareness
			Tempo discrimination	Melody discrimination	
			Beat synchronization	Melody production	
Ríos-López et al. (2019)	43	4–5	Beat synchronization		Phonological awareness
Steinbrink et al. (2019) (1)	54	5–7	Tempo discrimination	Pitch discrimination	Phonological awareness
			Rhythm discrimination	Melody discrimination	
			Rhythm reproduction		
Steinbrink et al. (2019) (2)	96	7–11	Tempo discrimination	Pitch discrimination	Reading comprehension
			Rhythm discrimination	Melody discrimination	Spelling
			Rhythm reproduction		Phonological awareness
Strait et al. (2011)	42	8–13	Rhythm discrimination	Melody discrimination	Reading ability
Tierney and Kraus (2013)	58	14–17	Beat synchronization		Reading ability
Tierney et al. (2017)	64	*M* = 18.0	Beat synchronization		Phonological awareness
			Rhythm reproduction		Reading ability
Woodruff Carr et al. (2014)	35	3–4	Beat synchronization	Melody discrimination	Phonological awareness
			Rhythm discrimination		Reading fluency
Yu et al. (2017)	511	*M* = 20.6	Rhythm discrimination	Pitch discrimination	Phonological awareness
Zhang et al. (2017)	54	8–10	Tempo discrimination	Pitch discrimination	Reading fluency
					Tonal sensitivity (Mandarin)
					Phonological awareness
Zuk et al. (2013)	45	6–8	Rhythm reproduction	Musical memory	Reading fluency
					Phonological awareness

*Note*. Rows correspond to individual studies, and columns correspond to study *N*s, age ranges in years, specifics of musical rhythm and tonal-melodic constructs measured, and reading constructs measured. When additional information is necessary about the *N*, specific descriptions are given in parentheses. When age range is not available, or not representative of the sample mean, the mean is provided in addition to the age range. Only musical constructs that were reported in relation to reading-related constructs are noted.

Converging evidence shows that a variety of rhythm-related skills (e.g., rhythm production, beat synchronization, memory for rhythm, timing/metrical perception) are robustly associated with building blocks of reading development. Musical rhythm perception and production are associated with phonemic skills such as word recognition and spelling (Douglas & Willatts, 1994), and letter-sound knowledge (Ozernov-Palchik et al., 2018; Ríos-López et al., 2019). Beat synchronization and rhythm discrimination are also associated with phonological awareness, rapid naming, and auditory working memory (Anvari et al., 2002; Moritz et al., 2013; Woodruff Carr et al., 2014). Musicality as a whole seems to be related to children’s early literacy skills at the start of formal education. Scores on a comprehensive music aptitude test (consisting of pitch, rhythm, and meter discrimination and production tasks) were associated with phonological awareness, nonword repetition, and rapid naming fluency in school-aged children even after controlling for key demographic and nonverbal cognitive variables (Degé et al., 2015).

Longitudinal investigations show that rhythm predicts later reading readiness, further suggesting a bootstrapping role of early musical ability for building blocks of reading development. Rhythm imitation (tapping a rhythmic sequence from memory) at entry to the first grade was predictive of children’s literacy skills as tested at the end of the same school year (Lundetræ & Thomson, 2018), and rhythm at kindergarten, predicted phonological awareness and word reading in the second grade (Moritz et al., 2013). David et al. (2007) found that rhythm skills in grade 1 predicted phonological awareness in grades 2 to 5. In a large sample (*N* = 600), rhythm reproduction in kindergarteners uniquely explained 14% of the variance in reading at grade 2, controlling for demographic factors, phonological abilities, visuo-spatial attention, and processing speed (Dellatolas et al., 2009), with children with lower SES backgrounds showing stronger rhythm-reading links. Taken together, these findings make a compelling case for exploring musical rhythm as a tool for reading interventions (Flaugnacco et al., 2014).

Evidence on rhythm-reading links also robustly converges at older stages of development. For example, González-Trujillo et al. (2014) found that in school-aged readers of Spanish, rhythm production/imitation (tapping from memory) was associated with reading fluency, and reading with accurate lexical stress patterns. Note that lexical stress is explicitly marked in Spanish words that diverge from canonical lexical stress patterns. Lê et al. (2020) similarly showed a link between rhythm skills and reading and spelling skills in third graders, after ruling out mediating contributions from both motor skills and phonological awareness.

The ability to analyze sound *sequences* seems specifically relevant for language/literacy. Grube et al. (2012) found associations between auditory perception of time and phonological skills, and between temporal sensitivity in musical sequences and phonological skills, across several standardized language and reading assessments. Further, a small study in adults (*n* = 26) representing a host of different native languages found that the ability to detect a regular rhythm within a highly irregular sequence (measured by a regularity detection threshold) predicts reading scores (here, rapid automatized naming) in their native language (Bekius et al., 2016).

In children, studies also show associations between tonal-melodic skills such as pitch processing, and phonemic awareness (Loui et al., 2011), nonword repetition (Flagge et al., 2016) and phonological awareness (Steinbrink et al., 2019). Pitch *production* skills are also correlated with phonemic awareness, controlling for IQ (Loui et al., 2011). Echoing their findings in the rhythm and temporal processing domain, auditory sequencing skills related to pitch and timbre correlated with phonological awareness abilities (Grube et al., 2012).

Musical listening and production (e.g., when playing an instrument) involves encoding/decoding hierarchically embedded information such as notes and chords, parallel to the encoding/decoding of phonemes, syllables, words, and sentences involved in reading. Indeed, two cohorts of preschoolers (American and Turkish) showed correlations between performance on a tone-deletion task with melodic excerpts and an equivalent phoneme deletion task with words and pseudowords (Peynircioğlu et al., 2002). Further, weaker performance at transcribing musical sequences from memory has been found to be associated with reading more slowly and less accurately (Zuk et al., 2013). Reading-related decoding skills in turn highly predicted performance on other linguistic tasks in a cognitive battery, consistent with the proposed role of musicality in language development (Figure 1).

Two studies shed light on the relationship between music and reading in the context of non-alphabetic languages (e.g., Chinese languages), where the building blocks of reading development involve other types of decoding than is typically measured in alphabetic languages. For example, melodic skills (but not rhythmic skills) were associated with semantic processing while reading Chinese (Yu et al., 2017). Further, English phonological awareness correlated with musical meter and pitch discrimination in English-Chinese bilinguals, mediating the relationship between musical perception and English reading. However, Chinese reading skills were not associated with musicality (Zhang et al., 2017).

##### Neural correlates of associations between musicality and literacy.

Both cortical and subcortical neural endophenotypes may be relevant for understanding music-reading links. Auditory brainstem responses (ABRs)—subcortical markers of auditory encoding—have been linked with speech perception skills in both children and adults. School-aged children showed enhanced ABRs to syllables in a predictable versus variable speech context, and ABR magnitudes correlated with both musical aptitude and word reading skills (Strait et al., 2011). These associations may exist even prior to literacy acquisition; for example, preschoolers’ beat synchronization abilities were associated with ABRs to syllables, and to behavioral preliteracy measures (e.g., phonological awareness, auditory short-term memory, and rapid automatized naming: Woodruff Carr et al., 2014). In adults, associations between efficient neural markers of auditory perception (e.g., ABRs to auditory clicks used in hearing screenings) are correlated with greater musical experience, as well as enhanced phonemic decoding (Tichko & Skoe, 2018).

As noted throughout this article, greater musical experience could itself be a function of both environmental and genetic influences. Similarly, evoked cortical responses to speech sounds have been associated with enhanced musical rhythm and reading skills (Tierney et al., 2017). Structural neural endophenotypes that might be involved in links between musicality and literacy include gray matter volume of primary auditory cortex structures. Specifically, larger relative gray matter volumes of Heschl’s gyrus compared to planum temporale, in both hemispheres, were correlated with reading and spelling skills in 7- to 9-year-old children (Seither-Preisler et al., 2014). Larger right Heschl’s gyrus volumes also correlated with more efficient processing of musical instrument sounds and tones (as measured by evoked P1 responses through magnetoencephalography, MEG), and strongly correlated with musical aptitude scores (Seither-Preisler et al., 2014). Beyond phonemic and phonological building blocks of reading, musical rhythm abilities are also associated with individual differences in electrophysiological correlates of reading (Fotidzis et al., 2018; Ríos-López et al., 2019).

#### Musicality and grammatical skills

##### Behavioral evidence: Musical abilities are correlated with morphosyntactic skills.

Morphosyntactic skills are foundational to language development across spoken and signed languages. Morphological manipulations allow children to form words from smaller units of meaning, and syntactic skills allow them to connect elements of language together to parse and produce sentences at multiple levels of syntactic hierarchy. These skills also make it possible to connect ideas within and across sentences, which facilitates other aspects of communication, e.g., expressing and comprehending ideas in speech and written language. Successful complex syntax acquisition enables social relationships, school-readiness, educational outcomes, and life skills (Brimo et al., 2017; Fujiki et al., 1999), and morphosyntactic disruptions are often a key feature of DLD. Table 5 summarizes studies examining associations between musicality and spoken grammar-related phenotypes.

**Table 5.** Studies reporting associations between musicality and morphosyntactic skills.

Study	*N*	Age	Rhythm construct	Tonal-melodic construct	Grammar-related construct
Cohrdes et al. (2016)	44	5–7	Rhythm reproduction	Melody reproduction	Receptive grammar
			Beat synchronization	Harmonic discrimination	
Gordon, Jacobs, et al. (2015)	25	5–7	Rhythm discrimination		Complex syntax
Gordon, Shivers, et al. (2015)	25	5–7	Rhythm discrimination		Expressive grammar
Lee et al. (2020) Exp 1	98	7–17	Rhythm discrimination		Receptive grammar
Lee et al. (2020) Exp 2	96	7–17	Rhythm discrimination		Receptive grammar
Nitin et al. (in press)	121	5–8	Rhythm discrimination		Expressive grammar
					Complex syntax
Politimou et al. (2019)	46	3–5	Rhythm discrimination	Pitch discrimination	Expressive/receptive grammar
			Tempo discrimination	Melody discrimination	
			Beat synchronization	Melody production	
Swaminathan and Schellenberg (2020)	91	6–9	Rhythm discrimination	Melody discrimination	Receptive grammar
				Musical memory (melodic)	
Woodruff Carr et al. (2014)	35	3–4	Beat synchronization		Expressive/receptive grammar
			Rhythm discrimination		

*Note*. Rows correspond to individual studies, and columns correspond to study *N*s, age ranges in years, specifics of musical rhythm and tonal-melodic constructs measured, and morphosyntactic constructs measured. When additional information is necessary about the *N*, specific descriptions are given in parentheses. When age range is not available, or not representative of the sample mean, means *M* are provided in addition to the age range. Only musical constructs that were reported in relation to grammatical/mophosyntactic constructs are noted.

Studies focused on preschoolers, school-aged children, and adolescents, find converging evidence for associations between musical rhythm traits and grammatical development. For example, in a sample of primary school-aged children (*N* = 25), Gordon, Shivers, et al. (2015) demonstrated that individuals who were more accurate at distinguishing between musical rhythm sequences, also performed more accurately on tasks that probed children’s ability to use particular morphosyntactic formations. Phonological awareness did not explain the association between musical rhythm and grammatical abilities, and in a follow-up study in a larger sample (*N* = 121), neither prosody perception skills nor working memory were found to mediate the relationship between musical rhythm and morphosyntactic skills (Nitin et al., in press). As seen in Table 5, there are as yet very few studies exploring the relationship between musical rhythm sensitivity and grammatical abilities.

These findings were extended by Swaminathan and Schellenberg (2020), who showed a strong association between musical beat perception and receptive grammar in ∼100 school-aged children performing the Test for Reception of Grammar (TROG: Bishop, 2003). The TROG is a grammatical comprehension task commonly used to measure grammatical impairments in children with DLD. This test, which asks children to point to the picture that corresponds with the sentence they hear, is commonly used to measure grammatical skill. Further, Politimou et al. (2019) showed that rhythm perception was associated with performance on a sentence imitation task (which encompasses both the receptive and expressive nature of grammar) in preschoolers. In keeping with these observations, preschoolers who had stronger abilities to synchronize to an external beat, had higher scores on tests of reading and sentence imitation (Woodruff Carr et al., 2014). Moreover, Lee et al. (2020) also found a correlation between rhythm discrimination and receptive grammar via a language comprehension task that required participants to identify the agent of the sentence, in a wider age range of participants (7- to 17-year-olds), while controlling for working memory, age, and musical training, showing that the relationship between musical rhythm sensitivity and grammar cuts across the developmental arc.

Melodic skills are also associated with grammatical abilities, and this relationship may be moderated by the richness of musical exposure and level of musical engagement in children’s home environments. For example, Politimou et al. (2019) found that children’s melody skills and degrees of music in the home were associated with grammar skills, particularly with tasks testing word structure awareness and sentence imitation. Moreover, in their study, the best model predicting grammar further included parental musicality measures, suggesting a potential genetic influence on music and grammar traits in children. Relatedly, *memory* for melodic patterns is associated with both musical rhythm and grammar ability (Swaminathan & Schellenberg, 2020).

##### Neural correlates of associations between musicality and grammatical skills.

A recent electroencephalography (EEG) study found that individual differences in children’s neural entrainment to the beat of rhythmic patterns (measured with evoked time-frequency analyses in beta and gamma bands) predicted variation in their expressive grammar abilities (Persici et al., 2022). These results are consistent with what many have proposed—that the overlap between musical rhythm and linguistic syntax is related to the processing of hierarchically organized syntax in language sharing common biology with the metrical aspect of rhythm processing (Asano et al., 2021; Fitch, 2017; Heard & Lee, 2020), for example, shared cognitive control circuitry in the frontal cortex and basal ganglia (Asano et al., 2021). However, see Fedorenko and Varley (2016) and Chen et al. (2021) for evidence that certain musical stimuli do not meaningfully activate language regions.

#### Musicality, second language learning, and tonal language development

##### Behavioral evidence: Musical abilities predict second language (L2) learning and tonal language development.

Second language (L2) and foreign language learning have long been heralded as a window into cognition. Findings about musicality from L2 and foreign language contexts converge with studies in native speakers. For example, musical aptitude (including specific abilities such as singing and harmonic discrimination) is associated with more accurate phonemic perception and production abilities (see Milovanov & Tervaniemi, 2011, for a review; Christiner & Reiterer, 2018), pronunciation (Turker et al., 2017) in a foreign language, and phonological abilities in an L2 (Slevc & Miyake, 2006). Further, musical aptitude predicts foreign language silent reading fluency better than other reading-related skills or auditory working memory (Foncubierta et al., 2020). Musicality was also associated with more accurate grammatical judgments of complex syntax in an artificial grammar learning task (Brod & Opitz, 2012). A school-based study of educational achievement found that Italian students’ grades in music classes were correlated with their grades in English and French classes at school (Picciotti et al., 2018). Given the relatively large sample size (*N* = 500), the naturalistic setting (the school system), and assessment of two foreign languages, this evidence suggests that individual differences in musical traits explain a proportion of the variation in foreign language learning achievement.

What about languages in which musical skills (e.g., pitch perception) are also language skills (e.g., lexical tone perception)? In this case, pitch skills might be a better predictor of language development than musical rhythm skills, in children acquiring tonal languages (Antoniou et al., 2015). However, pitch perception of auditory tones embedded in non-hierarchical contexts (e.g., non-musical/non-linguistic stimuli) did not predict Cantonese lexical tone perception (Wong & Cheng, 2020), suggesting that it is the hierarchical structures of musical rhythm that are specifically relevant for morphosyntactic parsing, rather than musical rhythm perception on the whole. This converges with other evidence about dissociations in tone versus lexical tone perception, e.g., only a subset of Mandarin Chinese speakers with congenital amusia show lexical tone perception deficits (Huang et al., 2015; Nan et al., 2010).

##### Neural evidence: The role of Heschl’s gyrus in musical and foreign language aptitude.

Structural brain imaging results have shown that individual differences in the morphology of the right Heschl’s gyrus are associated with both phonetic coding in an unknown foreign language, and with musical aptitude (Turker et al., 2017). Turker et al. argue that since the morphology of Heschl’s gyrus tends to be stable, musical and language abilities have relatively reduced flexibility and are privy to genetic influences or environmental influences that shape very early development (e.g., at the prenatal or very early postnatal stages). Thus, Heschl’s gyrus could be a key brain area of interest for examining genetic influences on phenotypic links between musical and foreign language aptitude. This result converges with other findings implicating Heschl’s gyrus in musicality-language links (e.g., Seither-Preisler et al., 2014, discussed in the Musicality, Reading Readiness, and Literacy section; Sutherland et al., 2012, discussed in the Building Blocks of Musicality and Language in Infancy section.

#### Building blocks of musicality and language in infancy

##### Behavioral evidence: Emerging musical and language abilities are correlated in infancy.

Infant-directed speech (Fernald et al., 1989; Leong et al., 2017), nursery rhymes, and infant-directed singing (Trehub & Trainor, 1998) are prevalent cross-culturally, and musical speech may in fact support language processing at the earliest stages of life. Accumulating evidence shows that the earliest stages of music and language development follow parallel tracks, and support culture-specific attunement to both music and speech (Brandt et al., 2012). Here, we synthesize infant studies examining correlations between early precursors of musical and language traits.

Infant studies contribute to understanding variability in musical and auditory traits that are present at the foundational stages of music and language development. For example, infants’ sensitivity to pitch (Leppänen et al., 2010) and to amplitude rise times of pure tones (Kalashnikova et al., 2019) predict later language-related traits such as phonological awareness, reading speed, and vocabulary development. Temporal auditory processing in infancy has also been shown to predict subsequent language production (Rocha et al., 2020), reading fluency (van Zuijen et al., 2012), and morphosyntactic skills (Höhle et al., 2014), consistent with previously discussed studies in children and adults. Höhle et al. (2014) found that infants’ rhythmic discrimination predicted sentence comprehension and morphological ability at age 5. Importantly, infants with family risk of language impairment were less able to discriminate rhythmic patterns, versus typically developing infants, consistent with studies in older children at-risk for language and reading disorders (see the Musicality and Developmental Disorders of Reading and Language section).

##### Neural evidence: Correlates of listening to tones, music, and sung speech.

A handful of neural investigations in infants have shown that individual differences in neural markers of temporal auditory processing predict language and reading outcomes later in development (Fava et al., 2014; François et al., 2017; van Zuijen et al., 2012). For example, newborns’ cortical responses to word form violations in sung speech streams predict vocabulary size at 18 months (François et al., 2017). Further, ERP responses to standard versus deviant inter-tone intervals in infancy are associated with reading fluency in later life (van Zuijen et al., 2012), and infants’ hemodynamic brain responses to both music and speech can be categorized into “high responder” and “low responder” groups based on neural engagement patterns (Fava et al., 2014).

Relationships between auditory processing in infancy and longitudinal language outcomes (e.g., Rocha et al., 2020) appear to persist in school-aged children. For example, Sutherland et al. (2012) found in a study of school-aged children, that changes in volume/structure in the auditory cortex (specifically Heschl’s gyrus) were associated with frequency modulation detection at 2 Hz—a measure of auditory perceptual sensitivity—and with reading and spelling abilities.

As suggested by the MAPLE framework, genetic investigations of neural endophenotypes associated with foundational musicality and language traits can help clarify the cascading biological and developmental effects of musicality-language links, in infancy and later in development.

#### Musicality and developmental disorders of reading and language

##### Behavioral evidence: Musical abilities are correlated with language abilities in developmental disorders of reading and language.

Developmental reading and language disorders (e.g., dyslexia; DLD) are primarily characterized by difficulties with written and/or spoken language abilities (e.g., reading, phonology, morphology, syntax). A recent theoretical framework synthesizes abundant evidence that individuals with such disorders also often show deficits in musical rhythm skills (Ladányi et al., 2020), and posits that atypical rhythm is a comorbid risk factor for childhood developmental speech and language disorders, potentially implicating shared genetic architecture (the atypical-rhythm risk hypothesis). Studies of musicality in language disorder contexts often emphasize group comparisons relative to typically developing individuals. Boorom et al. (2022) provide an extended discussion of the role of musical abilities and musical interventions in developmental language disorders. Here, we synthesize studies using individual differences approaches, which can provide more granular views of the entire spectrum of musical and language-related abilities represented in the population.

Studies of individual differences in cohorts of children with DLD have found associations between musical rhythm skills, and grammar skills, expressive and receptive language skills, and artificial grammar learning abilities. For example, an early study of children with DLD found associations between musical rhythm sensitivity and grammar skills across multiple standardized language tests (Weinert, 1992). This association also extended to an artificial grammar learning task with prosodically enriched stimuli. Across the spectrum of language ability (e.g., in children with DLD as well as in typically developing children), studies have found associations between musical beat perception and beat synchronization (i.e., ability to tap to a beat), and receptive and expressive language skills and reading skills (Corriveau & Goswami, 2009; Cumming et al., 2015), controlling for age and nonverbal IQ. Reading-related skills investigated include phonological awareness, word and nonword reading, reading comprehension, spelling, reading fluency, and nonword repetition.

There is also some evidence suggesting that tonal-melodic skills are associated with language abilities in preschoolers with DLD (Sallat & Jentschke, 2015). Here, morphosyntactic skills (measured by a sentence repetition task) were also found to correlate with performance on melody recognition, melody discrimination, and rhythmic-melodic perception tasks. Although, results should be interpreted cautiously because overall performance on the language task was at chance levels, and analyses did not control for demographic or cognitive factors.

Similarly, in the context of dyslexia, musical rhythm measures such as metrical sensitivity or rhythm reproduction are associated with reading-related skills, both in children (Flaugnacco et al., 2014; Huss et al., 2011; Overy, 2003) and in adults (Boll-Avetisyan et al., 2020). Forgeard et al. (2008) also reported significant correlations between melody discrimination abilities and phonemic awareness in a small sample of school-aged children with dyslexia. In a large-scale online cohort, time-based but not-pitch based congenital amusia was associated with self-reported dyslexia and speech disorder (Peretz & Vuvan, 2017), further highlighting the unique role that biological mechanisms supporting rhythm/temporal processing may play in driving musicality-language links.

#### Insights from the literature

Converging evidence thus suggests that musical abilities are highly relevant for our understanding of individual differences across the continuum of language abilities, in both typical development and language disorder contexts, and in both native and L2 contexts. Specifically, our literature synthesis highlighted the importance of multiple dimensions of musicality, including beat synchronization, rhythm and melody discrimination, and pitch discrimination abilities. Language-related phenotypes for which variance is explained in part by musical abilities include nonword repetition, complex syntax, sentence recall, speech perception-in-noise, speech rhythm discrimination, and L2 phonemic perception and production.

Studies examining associations between musical and language abilities have often examined and/or controlled for environmental factors such as family SES and degree of formal and/or informal musical training. However, recall from the MAPLE framework (Figure 2) that so-called environmental factors can themselves show significant heritability. For example, musicality can be influenced by gene-environment interactions (when genetic predispositions moderate environmental effects on a phenotype or endophenotype), or gene-environment correlations (when environments are selected or created in ways that are complementary to genetic predispositions). Therefore, the study of environmental effects can potentially be confounded by genetic factors (see Hart et al., 2021, for an expanded discussion of the issue). Future research on musicality-language associations should therefore aim to incorporate gene-environment correlations and gene-environment interactions into models where genetic and environmental data are available. Alternatively, where genetic information is not available, one option is to incorporate Hart et al.’s (2021) *familial control method* as a kind of genetic proxy. Here, the same trait is measured in parent and child and included as a covariate to estimate the effect of the rearing environment. Note that while this method can be included in some developmental work going forward, it may be currently difficult to account for genetic confounds in this way where child and adult-friendly measures of a music or language construct do not yet exist.

Prior studies have commonly included working memory as a measure of executive function (EF) skills, but without considering other aspects of EF (e.g., inhibitory control; attentional shifting). EF skills are implicated in both language (Gooch et al., 2016; Kapa & Erikson, 2020; Woodard et al., 2016) and musicality (Clayton et al., 2016; Moreno et al., 2011), and are some of the most heritable psychological traits (Friedman et al., 2008). In fact, cognitive control processes may have a particularly relevant role in musicality-language links, particularly for further understanding relationships between rhythm and grammar. For example, the coordinated hierarchical control hypothesis (Asano et al., 2021) points to coordination among the frontal cortex areas, for example, inferior frontal gyrus (IFG), dorsolateral prefrontal cortex (dlPFC), pre-supplemental motor area (pre-SMA), and the basal ganglia as a shared mechanism underlying musical rhythm and linguistic syntax processing, and specifically argues for the central importance of control processes such as inhibition, selection, and maintenance. While it is common to study EF in the context of both musical and language abilities, more robust investigations of individual differences in various measures of EF—perhaps especially inhibitory control—at behavioral, neural, and genetic levels, would allow for better developed predictions about musicality-language links across the lifespan.

A noteworthy pattern that emerged from the literature on musical abilities and reading-related skills was that current investigations generally focus on smaller building blocks of language (e.g., phonemes, syllables, words), but lack naturalistic reading tasks (e.g., reading sentences/passages; reading comprehension). Including these reading contexts in future studies of musicality-language links can shed light on the range of reading functions that may be supported by biological mechanisms involved in music and language, and may provide a novel focus for developing music-based reading interventions.

Further, many studies on reading highlighted that performance on nonword repetition tasks is robustly associated with both rhythmic and tonal-melodic musical abilities. Given this pattern, and studies suggesting that nonword repetition is a good proxy for assessing overall spoken language skills (i.e., higher-order language skills in the auditory-vocal modality), we argue that future models examining musicality-language links should consider nonword repetition as a key language-related trait of interest, beyond viewing it as a measure of phonological awareness or working memory in the manner that it has sometimes been designated in the phenotypic literature (e.g., Degé et al., 2015; Flagge et al., 2016). This point may be especially important for advancing the genetic study of musicality-language links within the MAPLE framework, as nonword repetition is an existing language-related phenotype in several large-scale genetic cohorts, and its heritability (Table 1) and genetic architecture (Eising et al., 2022) have been examined.

Our synthesis of the literature did not find a critical mass of studies focused on older adulthood reporting behavioral associations between musical and language abilities (although some studies have older adults in their broader adult samples). However, there are known associations between musical training and protective effects against age-related declines in speech perception outcomes in older adulthood (e.g., Bidelman & Alain, 2015), and interest in music-based interventions for healthy aging (James et al., 2020), age-related neurodegenerative conditions such as dementia or Alzheimer’s disease (Leggieri et al., 2019; Moreno-Morales et al., 2020), or stroke rehabilitation (Grau-Sánchez et al., 2020). The MAPLE framework takes a lifespan view, proposing that individuals “high” on a spectrum of musical *abilities* may show greater resilience to age-related decline in normal speech-language functions, e.g., SPIN or hearing acuity (Figure 1). Systematic efforts to understand individual differences in musicality-language associations in older adulthood, can create a strong foundation for a personalized approach to music-based interventions in the context of neurodegenerative conditions. Importantly, a lifespan approach allows us to probe genetic risk and resilience to speech-language outcomes based on individual differences in musicality.

Last, since musicality-language links are likely shaped by the diversity of both musical and language experiences, future research should pay special attention to capturing individuals’ language backgrounds, including years of simultaneous or sequential multilingual exposure; native tonal language backgrounds; and extent of engagement in language learning beyond native language(s). Capturing variability in both language *and* musical experiences will certainly provide a more complete picture of the relationship between musical and language abilities, and variation across the spectrum of these abilities. Ultimately, to map population-level relationships between genetic and neural mechanisms, environmental factors, and musicality and language traits, it will be imperative for research efforts to represent diverse cultures, languages, and musical systems.

Compared to the behavioral evidence showing covarying individual differences in musicality and language traits, our literature review found that studies testing for neural correlates of these associations were relatively sparse. Since the MAPLE framework proposes that the behavioral associations commonly found between musical and language abilities are partly driven by shared genetic influences, it is crucial to understand the neurobiological mechanisms through which these genetic influences are exerted, to meaningfully test the framework’s hypotheses. With the goal of mapping the relationships among genetics, neurobiology, and perception/cognition, future research should aim to integrate neuroscience and genetics approaches to test for potential neural endophenotypes of the (shared) genetic influences on musicality and language traits.

Potential neural endophenotypes involving brain *structures* are already available as part of large-scale data sets that include both neural and genetic information, such as the UK Biobank (Sudlow et al., 2015) or the Cohorts for Heart and Aging Research in Genomic Epidemiology (CHARGE) Consortium (Psaty et al., 2009). We can also define *functional* neural endophenotypes of interest based on available neuroimaging studies of musical and language processing, extracting genomic or gene expression data associated with functional activation data (Kong et al., 2020; Mekki et al., 2022). For example, Mekki et al. (2022) defined a language network based on an existing meta-analysis of fMRI-based language-related function, then used the network to explore the genetic architecture of resting state functional connectivity in the inferred language network, using individual genotypes from the UK Biobank (Sudlow et al., 2015). Therefore, even though *neuroimaging during a language task* was not conducted in UK Biobank participants, this triangulation among genes, brain, and behavior, makes it possible to gain insights into the genetic architecture of the relevant circuitry (although we do not yet know to what degree genetic associations with resting-state data reflect those that would be seen with task-based functional activation data from the same participants). Similarly, resources that focus on *developmental* trajectories, such as data from the Adolescent Brain Cognitive Development study (ABCD; Volkow et al., 2018) can be harnessed to understand neural mechanisms of musicality and language traits as they unfold during development.

In tandem, primary experimental research in the lab, with a focus on neuroimaging and electrophysiological approaches, can directly investigate structural, functional, and developmental correlates of musicality-language links at the intersection of specific traits of interest. In-lab approaches allow for “deeper” measurement or phenotyping in smaller samples, which are important for validating and qualifying findings from larger samples using the aforementioned triangulation methods.

## CONCLUSIONS

We introduced the Musical Abilities, Pleiotropy, Language, and Environment (MAPLE) framework, which integrates genetic and environmental explanations for the many behavioral associations between musicality and language skills. The MAPLE framework provides a bird’s eye view of individual differences that work together to explain covariation of musicality and language traits. Individual variation in shared genetic architecture between musicality and language may result in variation at the level of neurobiological development, structure, and function, along with effects on other biological systems potentially relevant for music and/or language abilities. This in turn explains covariance in cognitive and neural measures of music and language. Additionally, neurobiology and other biological systems supporting language traits are thought to be influenced by genetic and environmental factors that also influence musicality. We also introduced genetic approaches that can be leveraged in several ways to investigate associations between music and language, including to understand how musicality traits may function as risk factors or promotive factors for language development and speech processing abilities. In particular, since genetic studies in this area are still nascent, the behavioral and neural findings of interest described here help generate predictions that can be tested using a variety of genetics approaches, as groundwork is established in the genetics of musicality and language. Beyond fundamental discoveries of shared genetic architecture, incorporating genetic/genomic data into music cognition and language science approaches can also help disentangle genetic and environmental factors underlying musicality-language associations, and improve identification and intervention efforts for language-related disorders through a richer understanding of musicality correlates of language-related abilities.

As a basis for MAPLE’s predictions about shared genetic architecture, we synthesized patterns of findings in the behavioral and neural evidence linking musicality and language skills. By synthesizing the evidence for musicality-language links across multiple domains of language-related traits (i.e., speech perception; reading-related skills; grammatical skills), this literature review provides a timely overview as individual differences approaches gain traction in their ability to shed light on common biological origins of musicality and language. The predictions derived from the MAPLE framework can be tested by integrating behavioral and/or neural methods with genetics approaches, and highlight the incredible potential of multidisciplinary collaboration.

## ACKNOWLEDGMENTS

The authors wish to acknowledge Russ Beebe and Navya Thakkar for their assistance with illustrations; Jonathan Z. Liu and Gabija Zilinskaite for technical assistance; Nancy J. Cox for thoughtful discussions about the framework; and Miriam D. Lense, Anna V. Kasdan, and two anonymous reviewers for helpful feedback on earlier versions of the manuscript.

## FUNDING INFORMATION

Reyna L. Gordon, National Science Foundation (https://dx.doi.org/10.13039/100000001), Award ID: 1926794. Cyrille L. Magne, National Science Foundation (https://dx.doi.org/10.13039/100000001), Award ID: 1926786. Reyna L. Gordon, National Institutes of Health (https://dx.doi.org/10.13039/100000002), Award ID: R01DC016977. Reyna L. Gordon, National Institutes of Health (https://dx.doi.org/10.13039/100000002), Award ID: DP2 HD098859. Simon E. Fisher, Max Planck Society (no award ID). The content is solely the responsibility of the authors and does not necessarily represent the official views of the funders.

## AUTHOR CONTRIBUTIONS

**Srishti Nayak**: Conceptualization: Supporting; Data curation: Equal; Formal analysis: Lead; Investigation: Equal; Methodology: Equal; Project administration: Equal; Visualization: Supporting; Writing – original draft: Lead; Writing – review & editing: Lead. **Peyton L. Coleman**: Data curation: Equal, Formal analysis: Supporting; Investigation: Equal; Project administration: Supporting; Software: Lead; Visualization: Lead; Writing – original draft: Supporting; Writing – review & editing: Supporting. **Enikő Ladányi**: Formal analysis: Supporting; Methodology: Supporting; Writing – original draft: Supporting; Writing – review & editing: Supporting. **Rachana Nitin**: Formal analysis: Supporting, Methodology: Supporting; Writing – original draft: Supporting; Writing – review & editing: Supporting. **Daniel E. Gustavson**: Formal analysis: Supporting; Investigation: Supporting; Methodology: Supporting; Writing – original draft: Supporting; Writing – review & editing: Supporting. **Simon E. Fisher**: Funding acquisition: Equal; Methodology: Supporting; Project administration: Equal; Writing – original draft: Supporting; Writing – review & editing: Equal. **Cyrille L. Magne**: Formal analysis: Supporting; Funding acquisition: Equal; Investigation: Supporting; Methodology: Supporting; Writing – original draft: Supporting. **Reyna L. Gordon**: Conceptualization: Lead; Formal analysis: Equal; Funding acquisition: Lead; Methodology: Equal; Project administration: Lead; Supervision: Lead; Visualization: Supporting; Writing – original draft: Supporting; Writing – review & editing: Equal.

## TECHNICAL TERMS

Musicality: The myriad ways in which humans interact with music, including aptitude, skills, engagement, listening, and enjoyment (Honing, 2018).
Phenotype: A trait of interest that can be measured or observed (e.g., having blue eyes, or a given score on a quantitative measure of rhythm abilities).
Polygenic pleiotropy: When the same sets of genetic variants make contributions to two or more distinct complex traits, pointing to shared genetic architecture.
Endophenotype: A specific biomarker associated with an illness/trait in the population that is heritable and exists whether or not the illness/trait is active (Gottesman & Shields, 1972).
Gene expression: The process by which DNA information is used to assemble RNA molecules and proteins through transcription and translation.
Heritability: An estimate of the proportion of trait variance accounted for by variation at the genetic level in a particular population, under specific environmental circumstances.
Affordances: Properties of the environment or neurocognitive makeup (e.g., music perception skills) that allow individuals to carry out actions (e.g., frequent music listening).
Genome-wide association study (GWAS): A systematic, comprehensive screen of genetic variants at many different sites across all chromosomes of a genome, testing each variant for association with a trait of interest.
Polygenic scores (or Polygenic Risk Scores): A number reflect a person’s estimated genetic predisposition for a trait, by combining the estimated effects of genetic variants at different loci across the genome.
Enriched: Overrepresentation of a biological function in the genetic architecture of a trait, compared to chance.
