# Supplementary material for: The Musical Abilities, Pleiotropy, Language, and Environment (MAPLE) Framework for Understanding Musicality-Language Links Across the Lifespan
Source: Neurobiol Lang (Camb). 2022 Dec 16;3(4):615–64. doi: 10.1162/nol_a_00079 (PMC9893227; doi:10.1162/nol_a_00079)
Supplement: Supplementary file 1 [file nol-3-4-615-s001.docx]

**Supporting Information**

**The Musical Abilities, Pleiotropy, Language, and Environment (MAPLE) Framework for Understanding Musicality-Language Links Across the Lifespan**

Srishti Nayak,^1,2^ Peyton L. Coleman,^1, 12^ Eniko Ladányi,^1,11^ Rachana Nitin,^1,3^ Daniel E. Gustavson,^4,5^ Simon E. Fisher,^6,7^ Cyrille L. Magne,^2,8^ Reyna L. Gordon^1,3,4,9,10,12^

^1^Dept. of Otolaryngology - Head & Neck Surgery, Vanderbilt University Medical Center, Nashville, TN, USA
^2^Dept. of Psychology, Middle Tennessee State University, Murfreesboro, TN, USA
^3^Vanderbilt Brain Institute, Vanderbilt University, Nashville, TN, USA
^4^Vanderbilt Genetics Institute, Vanderbilt University Medical Center, Nashville, TN, USA
^5^Dept. of Medicine, Vanderbilt University Medical Center, Nashville, TN, USA
^6^Language and Genetics Department, Max Planck Institute for Psycholinguistics, Nijmegen, The Netherlands
^7^Donders Institute for Brain, Cognition and Behaviour, Radboud University, Nijmegen, The Netherlands
^8^PhD Program in Literacy Studies, Middle Tennessee State University, Murfreesboro, TN, USA
^9^Curb Center for Art, Enterprise, & Public Policy, Vanderbilt University, Nashville, TN, USA
^10^Vanderbilt Kennedy Center, Vanderbilt University Medical Center, TN, USA
^11^Dept. of Linguistics, Potsdam University, Potsdam, Germany
^12^ Vanderbilt University School of Medicine, Vanderbilt University, TN, USA

**Author Note**

Srishti Nayak
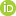
 [https://orcid.org/0000-0001-6581-3510](https://orcid.org/0000-0001-6581-3510%20)

Peyton L. Coleman
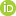
 <https://orcid.org/0000-0001-5388-6886>

Enikő Ladányi
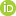
 <https://orcid.org/0000-0003-2853-682X>

Rachana Nitin
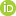
 <https://orcid.org/0000-0002-3331-1580>

Daniel E. Gustavson
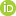
 <https://orcid.org/0000-0002-1470-4928>

Simon E. Fisher
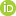
 <https://orcid.org/0000-0002-3132-1996>

Cyrille L. Magne
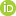
 <https://orcid.org/0000-0003-4943-9244>

Reyna L. Gordon
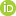
 <https://orcid.org/0000-0003-1643-6979>

Correspondence concerning this article and supporting information should be addressed to: Srishti Nayak, PhD [srishti.nayak@vumc.org](mailto:srishti.nayak@vumc.org) and Reyna Gordon, PhD [reyna.gordon@alumni.usc.edu](mailto:reyna.gordon@alumni.usc.edu), Vanderbilt Music Cognition Lab, 1215 21^st^ Ave. South, Medical Center East, Vanderbilt University Medical Center, Nashville, TN.

# Details of Bibliometric Analysis

State-of-the-field visualizations (manuscript Fig. 3 and Fig. 4) were created using VOSviewer (van Eck & Waltman, 2010). This program uses a specific literature search to visualize the prevalence of various terms within the literature, as well as how those terms are linked together. We used the following search on PubMed: “((music*[Title/Abstract]) OR (rhythm*[Title/Abstract]) OR (tonal*[Title/Abstract]) OR (melod*[Title/Abstract])) AND ((language[Title/Abstract]) OR (prosod*[Title/Abstract]) OR (gramma*[Title/Abstract]) OR (speech[Title/Abstract]) OR (linguist*[Title/Abstract]) OR (synta*[Title/Abstract])) NOT (heart) NOT (cardi*) NOT (sinus*) NOT (atrial) NOT (circadian)”, which yielded 5,013 results.

We first visualized the state of the field with default VOSviewer settings for bibliographic mapping (60% of the most relevant terms, and a maximum of 1,000 lines visualized) for greater clarity (Fig. 3 in manuscript). “Relevant terms” are based on a relevance score calculated by the program, which analyzes the number of times a term is used across all papers. The top 40% “most relevant” terms are filtered out to avoid capturing irrelevant words like “experiment” or “science”. The number of lines corresponds to how many of the links between terms are shown in the figure, and the strongest links (i.e., terms that occur together in the published literature more often) are prioritized. Terms must have occurred in at least 10 publications to be included.

To examine direct links between terms of special interest here (“individual differences”, Fig. 4; “musical training”, Fig. 4), we increased both the relevant terms included to 85%, and the number of lines (links) visualized. For the queries related to “individual differences” and “music training”, 10,000 lines were included, whereas for “musical training”, 5,000 lines were captured. This is evidence that “individual” and “differences” are not used together enough to be considered a single term until the relevance is raised to 85%. Lines connecting “individual differences” to other terms did not appear until 10,000 lines were enabled, which is further evidence that the term is not widely used in publications. Figure S.1 below displays all possible terms (100% relevance).

The term “individual differences” occurred 84 times in this literature search. In addition to the terms “music training”, “musical training”, and “formal musical training” occurring 360 times as reported in the manuscript, the addition of terms “formal training” (*n* =13) and “training effect (*n* = 15) raise the number of occurrences from 360 to 388. While this analysis could not determine if these terms were published in 360 separate papers, or if some published papers use many of these terms (e.g., a paper studying both musical training and individual differences), it is indicative of the relatively higher prevalence of the musical training frameworks compared to individual differences-based ones.

**Figure S.1**


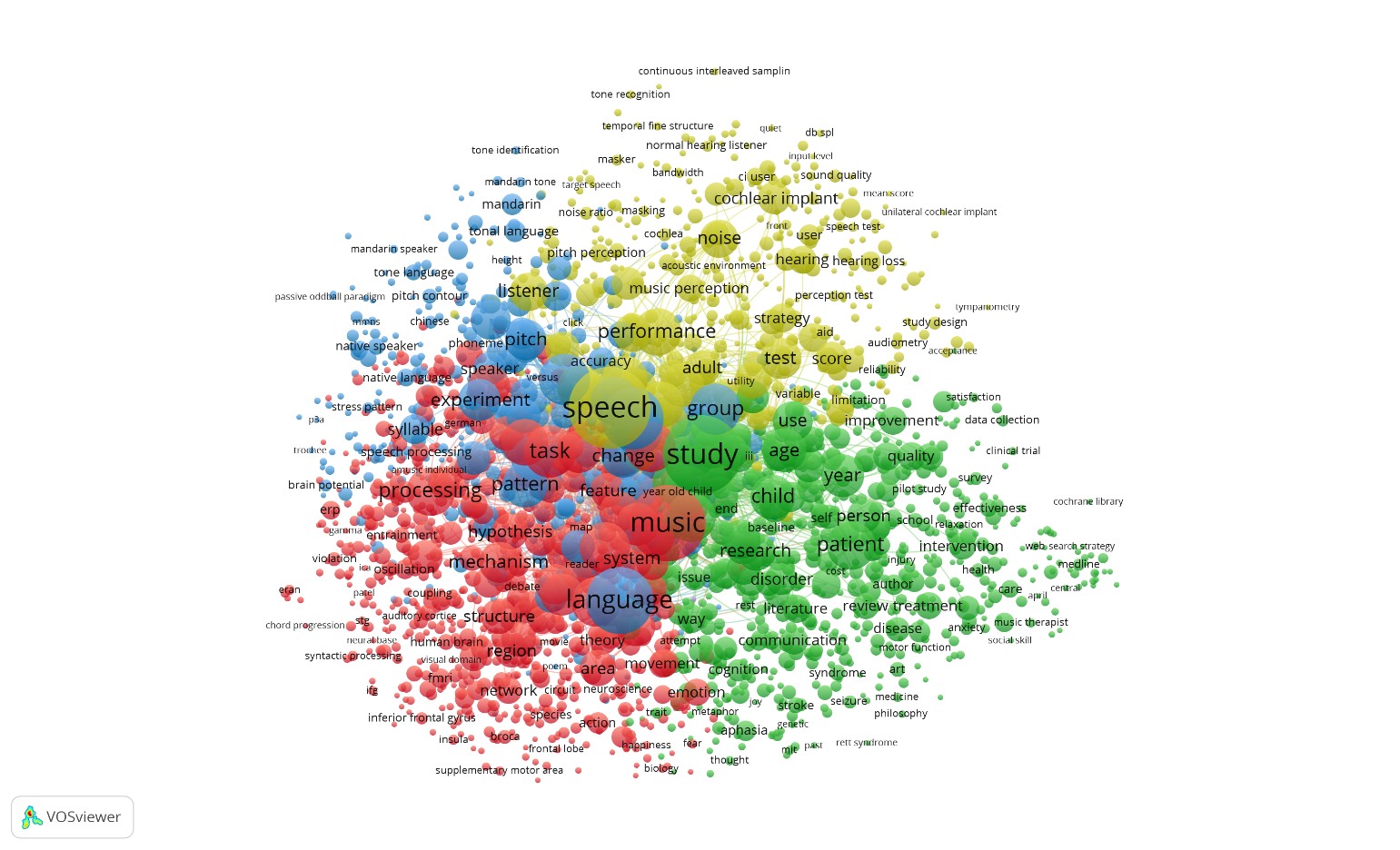
*Visualization of All Relevant Terms with Bibliographic Mapping*

*Note.* Figure showing bibliographic mapping based on music and language science related search terms, when 100% of the most relevant terms are visualized.

**Glossary of Key Terms**

1. ***Affordances:*** Properties of the environment that allow or enable an individual or organism to carry out certain actions. Here, used also to describe properties of the neurocognitive makeup (or “environment”) of individuals. For example, someone with stronger music perception skills may enjoy certain aspects of music more, influencing them to listen to music more frequently.
2. ***Assortative mating***: the idea that individuals choose partners based on genetic compatibilities, leading to amplification of certain traits within families.
3. ***Decoding***: the ability to break written words down into sounds or syllables based on the phonemic representations of your language to support word recognition.
4. ***Developmental dyslexia***: a neurodevelopmental disorder in which there are difficulties extracting meaning from written text.
5. ***Developmental language disorder (DLD)***: a disorder in which there are difficulties in language production and/or comprehension. As recommended by Bishop (2017), this term is now used instead of specific language impairment (SLI), as it was referred to in some of the currently reviewed studies.
6. ***Enrichment:*** when particular biological functions are overrepresented in the genetic architecture of a particular trait as compared to what would be expected based on chance, for example as discovered through gene-set enrichment analysis (Subramanian et al., 2005)
7. ***Endophenotype***: a specific biomarker that, as outlined by Gottesman & Shields (1972), (a) is associated with illness/trait in the population, (b) is heritable, (c) is primarily state-independent (exists whether or not the illness/trait is active), (d) co-segregates with illness/trait within families, and (e) the biomarker found in affected family members exists in nonaffected family members at a higher rate than in the general population.
8. ***Gene***: a distinct sequence of nucleotides that is translated into proteins or other molecules necessary for cell function.
9. ***Gene expression***: the process by which DNA information is used to assemble RNA molecules and proteins through transcription and translation.
10. ***Gene x environment interactions***: when two genotypes respond to an environment in different ways (e.g., having a different response to music lessons depending on whether or not you have genes associated with better musical ability).
11. ***Gene regulation***: the process that controls the timing, location and amount in which genes are expressed. Gene regulation is carried out through various mechanisms, including through regulatory proteins and chemical modification of DNA, and can be affected by environmental influences.
12. ***Genome wide association study (GWAS)***: a systematic and comprehensive screen of genetic variants at a great many different sites across all chromosomes of the genome, testing each variant for association with a trait of interest (either by comparing cases to controls, or analyzing the relationship with a quantitative measure). Since effect sizes are small, large cohort of thousands, or tens of thousands, of people are needed to ensure adequate statistical power in such studies.
13. ***Heritability***: an estimate of the proportion of trait variance that may be accounted for by variation at the genetic level in a particular population of interest, under a particular set of environmental circumstances.
14. ***Morphosyntactic skills***: skills relating to how words are formed and how the order of words results in understandable phrases and clauses.
15. ***Musicality***: The myriad ways in which humans interact with music, including aptitude, skills, engagement, listening, enjoyment, and so on (Honing, 2018).
16. ***Niche-picking***: the idea that individuals, including children, have a strong role to play in constructing their environments in ways that complement their genetic predispositions (Scarr, 1996; Scarr & McCartney, 1983).
17. ***Phenotype:*** a trait of interest that can be measured or observed (e.g., having blue eyes, or a given score on a quantitative measure of rhythm abilities).
18. ***Phonological awareness***: awareness or sensitivity to the syllabic and sub-syllabic units of language. This includes the ability to manipulate the sound structure of words, and is a crucial skill for learning to become a proficient reader.
19. ***Pleiotropy***: when one gene influences two or more distinct phenotypes
20. ***Polygenic***: when phenotypic variance in a trait is influenced by combined effects of genetic variants at many different genomic loci.
21. ***Polygenic pleiotropy***: when the same sets of genetic variants make contributions to two or more distinct complex traits, pointing to shared genetic architecture.
22. ***Polygenic scores (or Polygenic Risk Scores)***: a number that reflects a person’s estimated genetic predisposition for a trait of interest, by combining the estimated effects of many genetic variants at different loci across the genome.
23. ***Prosody***: the patterns of stressed and unstressed syllables in language, as well as the intonation of these stress patterns.
24. ***Reading fluency***: the speed and accuracy in which individuals can read written text aloud with appropriate expression.
25. ***Rhythm***: temporal arrangement of sound, including but not limited to: beat (the main driving pulse), tempo (speed), metricality (the arrangements of beats, e.g., in groups of 3 in a waltz), as well as unique combinations of long and short durations of sounds in any musical piece.
26. ***Single Nucleotide Polymorphism (SNP):*** a commonly occurring variation among individuals, at a single position in a DNA sequence.
27. ***Speech-perception-in-noise (SPIN)***: the ability to discriminate speech from background noise.
28. ***Tonal-melodic***: related to the tonal arrangement of sound, including but not limited to: pitch (the frequency of a single note), melody (the combination of many pitches over time), and harmony (the combination of many pitches at one time).

**References**

Bishop, D. V. (2017). Why is it so hard to reach agreement on terminology? The case of developmental
language disorder (DLD). *International Journal of Language & Communication Disorders*, *52*(6), 671-680.

Gottesman, I. I., & Shields, J. (1972). Schizophrenia and genetics. A twin study vantage point. In *ACAD. PRESS, NEW YORK, NY*.

Honing, H. (2018). On the biological basis of musicality. *Annals of the New York Academy of Sciences*, *1423*(1), 51-56.

Scarr, S. (1996). How people make their own environments: Implications for parents and policy makers. *Psychology, Public Policy, and Law*, *2*(2), 204.

Scarr, S., & McCartney, K. (1983). How people make their own environments: A theory of genotype → environment effects. *Child Development*, 424-435.

Subramanian, A., Tamayo, P., Mootha, V. K., Mukherjee, S., Ebert, B. L., Gillette, M. A., Paulovich, A., Pomeroy, S. L., Golub, T. R., Lander, E. S., & Mesirov, J. P. (2005). Gene set enrichment analysis: A knowledge-based approach for interpreting genome-wide expression profiles. *Proceedings of the National Academy of Sciences*, *102*(43), 15545–15550. <https://doi.org/10.1073/PNAS.0506580102>

Van Eck, N., & Waltman, L. (2010). Software survey: VOSviewer, a computer program for bibliometric mapping. *Scientometrics*, *84*(2), 523-538.
